# Hepatic ketone body regulation of renal gluconeogenesis

**DOI:** 10.1016/j.molmet.2024.101934

**Published:** 2024-04-09

**Authors:** Ryo Hatano, Eunyoung Lee, Hiromi Sato, Masahiro Kiuchi, Kiyoshi Hirahara, Yoshimi Nakagawa, Hitoshi Shimano, Toshinori Nakayama, Tomoaki Tanaka, Takashi Miki

**Affiliations:** 1 Department of Medical Physiology, Chiba University, Graduate School of Medicine, Chiba 260-8670, Japan; 2 Research Institute of Disaster Medicine (RIDM), Chiba University, Graduate School of Medicine, Chiba 260-8670, Japan; 3 Laboratory of Clinical Pharmacology and Pharmacometrics, Chiba University, Graduate School of Pharmaceutical Sciences, Chiba 260-8670, Japan; 4 Department of Immunology, Chiba University, Graduate School of Medicine, Chiba 260-8670, Japan; 5 Division of Complex Biosystem Research, Department of Research and Development, Institute of Natural Medicine, University of Toyama, Toyama 930-0194, Japan; 6 Department of Endocrinology and Metabolism, Institute of Medicine, University of Tsukuba, Ibaraki 305-8575, Japan; 7 Department of Molecular Diagnosis, Chiba University, Graduate School of Medicine, Chiba 260-8670, Japan

**Keywords:** Renal gluconeogenesis, Ketone bodies, Acid-base homeostasis, Glucose metabolism

## Abstract

**Objectives:**

During fasting, liver pivotally regulates blood glucose levels through glycogenolysis and gluconeogenesis. Kidney also produces glucose through gluconeogenesis. Gluconeogenic genes are transactivated by fasting, but their expression patterns are chronologically different between the two organs. We find that renal gluconeogenic gene expressions are positively correlated with the blood β-hydroxybutyrate concentration. Thus, we herein aim to investigate the regulatory mechanism and its physiological implications.

**Methods:**

Gluconeogenic gene expressions in liver and kidney were examined in hyperketogenic mice such as high-fat diet (HFD)-fed and ketogenic diet-fed mice, and in hypoketogenic PPARα knockout (PPARα^−/−^) mice. Renal gluconeogenesis was evaluated by rise in glycemia after glutamine loading *in vivo*. Functional roles of β-hydroxybutyrate in the regulation of renal gluconeogenesis were investigated by metabolome analysis and RNA-seq analysis of proximal tubule cells.

**Results:**

Renal gluconeogenic genes were transactivated concurrently with blood β-hydroxybutyrate uprise under ketogenic states, but the increase was blunted in hypoketogenic PPARα^−/−^ mice. Administration of 1,3-butandiol, a ketone diester, transactivated renal gluconeogenic gene expression in fasted PPARα^−/−^ mice. In addition, HFD-fed mice showed fasting hyperglycemia along with upregulated renal gluconeogenic gene expression, which was blunted in HFD-fed PPARα^−/−^ mice. *In vitro* experiments and metabolome analysis in renal tubular cells showed that β-hydroxybutyrate directly promotes glucose and NH_3_ production through transactivating gluconeogenic genes. In addition, RNA-seq analysis revealed that β-hydroxybutyrate-induced transactivation of *Pck1* was mediated by C/EBPβ.

**Conclusions:**

Our findings demonstrate that β-hydroxybutyrate mediates hepato–renal interaction to maintain homeostatic regulation of blood glucose and systemic acid-base balance through renal gluconeogenesis regulation.

## Highlights

• Renal gluconeogenic gene expression is transactivated under ketogenic conditions.
• Activated renal gluconeogenesis contributes to fasting hyperglycemia in obese mice.
• β-hydroxybutyrate directly transactivates renal gluconeogenic gene expression.
• β-hydroxybutyrate supplementation ameliorates fasting hypoglycemia in PPARα^−/−^ mice.
• β-hydroxybutyrate is a regulator of glucose and acid-base homeostasis in kidney.

## Graphical abstract

**Figure:**
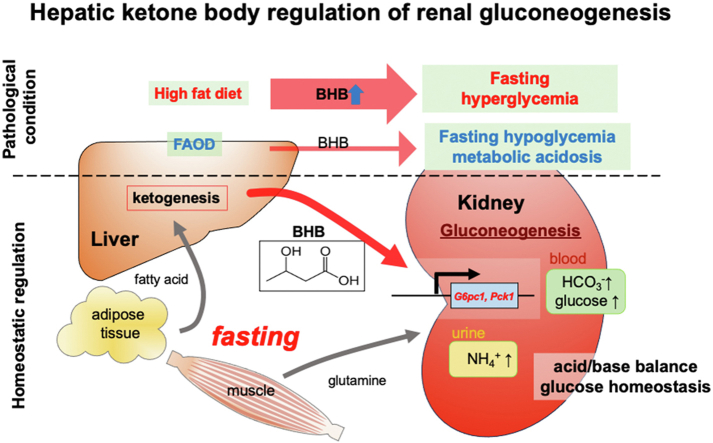

## 1 Introduction

Gluconeogenesis is a crucial metabolic pathway in maintaining blood glucose levels during fasting by generating glucose from non-carbohydrates including amino acids, lactate, pyruvate, and glycerol. Gluconeogenesis is induced by anti-insulin hormones including glucagon, glucocorticoids, epinephrin, and growth hormone via transactivation of rate-limiting gluconeogenic enzymes such as G6Pase and PEPCK, which are coded in *G6pc1* and *Pck1*, respectively [1]. Expression of these enzymes occurs only in certain cell types found in hepatocytes, renal proximal tubule cells, and intestinal epithelial cells, which are essential for systemic blood glucose regulation in fasting conditions. The contribution of extrahepatic gluconeogenesis in the maintenance of blood glucose levels has been reported in several studies [[2], [3], [4], [5], [6], [7]]. During post-absorptive phase, renal gluconeogenesis is induced and accounts for ∼40% of endogenous gluconeogenesis [3]. Notably, gluconeogenic genes expressed in the kidney are reported to be further activated in prolonged fasting while those in the liver are downregulated [4]. Gerich et al. has reported that endogenous glucose production by kidney and liver is cooperative, for which they propose the term *hepatorenal reciprocity* [[5], [6], [7]]*.* While several studies indicate that gluconeogenesis is controlled through transcriptional regulation of *G6pc1* and *Pck1* in the liver [1], the regulatory mechanism of gluconeogenesis in the kidney remains to be elucidated.

In type 2 diabetes mellitus (T2DM) patients, in addition to impaired insulin secretion and impaired insulin-induced glucose uptake, enhanced endogenous glucose production (EGP) is known to contribute to the development of hyperglycemia [8]. Michael et al. found that impaired hepatic insulin signaling can cause severe hyperglycemia and glucose intolerance through enhanced hepatic glucose production using hepatocyte-specific insulin receptor knockout (LIRKO) mice [9]. In addition, several studies suggest that increased fasting EGP through gluconeogenesis, but not through glycogenolysis, contributes to fasting hyperglycemia [[10], [11], [12]]. Interestingly, Samuel et al. previously reported that hepatic gluconeogenic gene expression is not upregulated in obese diabetic mice or obese diabetic patients despite their fasting hyperglycemia [13], which suggests a contribution of extrahepatic glucose production to hyperglycemia in obesity. Moreover, Meyer et al. have shown that renal glucose release in postabsorptive and postprandial states is abnormally upregulated in diabetic patients, increasing glucose release by kidney to a level comparable to that by liver [14,15]. Thus, increased renal gluconeogenesis might well contribute to hyperglycemia in diabetic patients on restricted diets, raising the possibility of its amelioration as a novel therapeutic option for T2DM.

Recent studies have found that renal gluconeogenesis is downregulated by insulin signaling, suggesting the involvement of insulin resistance in increased renal glucose production in T2DM patients [16,17]. However, the coordination of hepatic and renal gluconeogenesis cannot be explained by alteration in blood hormone level since gluconeogenesis in the two organs is not concurrent. Sasaki et al. [16] also suggest that decreased glucose influx into proximal tubules (PTs) upregulates renal gluconeogenesis in fasting state, while there is a vicious cycle in worsening the fasting hyperglycemia in T2DM patients in which renal gluconeogenesis is upregulated despite high glucose influx into PTs in fasting state [18]. Therefore, other, unknown mechanisms are involved in coordinating gluconeogenesis in liver and kidney. In the present study, we demonstrate that upregulated hepatic ketogenesis elicits concurrent upregulation of renal gluconeogenic gene expression via blood β-hydroxybutyrate (BHB), a ketone body generated by the liver in fasting conditions.

## 2 Materials and methods

### 2.1 Reagents

Sodium β-hydroxybutyrate (Na BHB) and 1,3-butandiol (1,3-BD) were purchased from Wako pure chemical (Osaka, Japan). BPTES, pimozide and withaferin A were purchased from Selleck Biotechnology (Tokyo, Japan).

### 2.2 Animal experiments

C57BL/6 mice (male, 12–20 weeks age) were used in this study. PPARα knockout (PPARα^−/−^) mice were purchased from Jackson Laboratory (Bar Harbor, ME). Insulin receptor mutant (*Insr*^*P1195L/+*^, referred to as mIR) mice were generated as described previously [19]. The mice were housed in a climate-controlled room (temperature, 23 ± 3 °C; humidity of 55 ± 15 %), and a 12-hr light/dark cycle. The mice were fed standard laboratory chow (CE-2; 12.1%kcal from fat, Clea Japan Inc., Tokyo, Japan), high-fat diet (HFD) or ketogenic diet (KD) (D12492; 60%kcal from fat or D10070801; 90%kcal from fat, Research Diets Inc., NJ) *ad libitum*. HFD was fed for 12 weeks, and KD was fed for 2 weeks starting at 8 weeks of age. A course of fasting for up to 48 h (0, 6, 12, 18, and 48 h) was started from ZT0. In other experiments, 16-hr fasting was conducted starting at ZT12. Sampling in the fed state was performed at ZT0 to avoid an apparent effect of fasting. For the glutamine tolerance test, 16-hr fasted mice were administered intraperitoneally with 250 mg/kg glutamine; blood glucose (BG) levels were monitored at the indicated time points. BPTES was pre-administered (10 mg/kg, *i. p.*) 1 h before starting a test. BG was measured as described previously [19]. Blood BHB was measured using Precision Exceed Pro (Abbot, Abbott Park, IL). Hepatic glycogen content was measured using a kit from BioVision (Waltham, MA). 20% (v/v) 1,3-BD was orally administered (10 μL/g BW) 2 h before sampling. Kidney and liver were harvested for the following analyses. Renal cortex was separated for qRT-PCR analysis.

To measure renal venous glucose output, we performed gonadal vein cannulation in anaesthetized mice. Peripheral blood was collected by the cannulation of the carotid artery.

Under anesthesia, 1,3-BD (20% (v/v) dissolved in saline) or vehicle was intraperitoneally administrated (10 μL/g BW), and peripheral and renal venous blood was collected for glucose and BHB concentrations before and after administration (30 min and 1 h).

All animal experiments were approved by the Animal Care Committee of Chiba University.

### 2.3 Cell culture

HK-2 cells, a human-derived PT cell line, were purchased from ATCC (Manassas, VA, #CRL-2190). The cells were cultured in D-MEM/F12 medium (Wako, Osaka, Japan) containing 10% FBS and penicillin/streptomycin. To stimulate gluconeogenesis, confluent HK-2 cells were cultured in conditions of increased oxygen supply by shaking at 60 rpm. The cells were treated with Na BHB in serum-free HBSS (+) for 3 h. Total RNA was extracted using RNeasy Mini kit (Qiagen, MD) and cDNA was synthesized for real-time qRT-PCR analysis. siRNA against human *CEBPB* was generated by Ambion (Thermo Fischer Inc. MA). Scramble or *CEBPB* siRNA was transfected using Lipofectamine RNAiMAX reagent (Thermo Fischer Inc. MA) following the manufacture's protocol. Seventy-two hours after transfection, total RNA was extracted for qRT-PCR analysis. For measurement of glucose production, cells were cultured with Krebs-Ringer-buffer (KRB) (135 mM NaCl, 3.6 mM KCl, 2 mM NaHCO_3_, 0.5 mM NaH_2_PO_4_, 0.5 mM MgCl_2_, 1.5 mM CaCl_2_, and 10 mM Hepes, pH7.4). After Na BHB treatment, the supernatant was collected for measurement of glucose, and the cells were subjected to NaOH lysis for protein extraction. The glucose concentration in medium was measured by Glucose-Glo assay (Promega Corp., WI). The values were normalized by the protein concentration.

### 2.4 Real-time qRT-PCR analysis

Quantitative real-time PCR was performed under standardized protocol as previously described [19]. The primers used are shown in Supplementary Table S1.

### 2.5 Immunofluorescent analysis and western blot analysis

Immunostaining of kidney tissues and western blot analyses were performed as previously described [20]. Details are provided in the supplementary materials. Antibodies used in this study are shown in Supplementary Table S2.

### 2.6 Isolation of proximal tubules and assays for detection of glucose and ammonium production

Male 12- to 18-week-old mice were sacrificed and kidneys were perfused by PBS containing Dynabeads M450 Tosyl-activated (Veritas Corp., Tokyo, Japan) for elimination of glomeruli from tubular suspension, as previously reported [21]. Kidneys were immediately harvested and washed in sterile ice-cold HBSS. Renal cortices were dissected in ice-cold dissection solution (DS) (HBSS containing 10 mM glucose, 5 mM glycine, 1 mM alanine, 15 mM HEPES, pH 7.4) and minced into fragments of 1 mm^3^. They were then transferred to collagenase solution (DS with 0.1% type-1 collagenase) and digested for 30 min at 37 °C. The digested tissue was sieved through two nylon sieves (200 μm, and 70 μm). The PTs remaining in the 70 μm sieve were resuspended by warm DS (37 °C) containing 1% BSA. The suspensions were centrifuged for 5 min at 500×*g*, washed and then resuspended into DMEM/F12 medium supplemented with 10% FBS. Glomeruli were completely eliminated from the tubular suspension using Magnet.

For glucose and ammonium production assay, PTs were resuspended into KRB buffer supplemented with 2.5 mM glutamine and 0.1% BSA. The solution was gassed with 95% O_2_/5% CO_2_ before use. PTs were cultured in a CO_2_ incubator (37 °C, 5% CO_2_) with shaking at 60 rpm by an orbital shaker for the indicated times. Inhibitors [pimozide (10 μM), and withaferin A (10 μM)] were added into the medium. The suspensions were then centrifuged for 10 min at 1,500×*g* and supernatants were collected for analysis. The pellets were lysed with 1 N NaOH and the lysate was used for determination of protein concentration. The glucose concentration in medium was measured using Glucose-Glo assay (Promega Corp., WI). The ammonia concentration in medium was measured by Amplite Colorimetric Ammonia Assay Kit (AAT Bioquest, Inc., CA). Values were normalized by protein concentration.

### 2.7 RNA-seq analysis

Vehicle or 20% (v/v) 1,3-BD was orally administered to the mice. After 2 h, the blood BHB concentration was measured, and the mice were sacrificed for analysis. The renal cortex was excised, and renal PT suspensions were prepared as mentioned above. Total RNA was isolated using RNeasy Mini plus kit (Qiagen, MD). (for detailed protocols for RNA-sequencing, please refer to the Supplementary Materials).

### 2.8 Metabolome analysis

Metabolome analysis was performed by capillary electrophoresis time-of-flight mass spectrometry (CE-TOF-MS). Cellular extracts were prepared as described previously [22]. Briefly, HK-2 cells were plated at a density of 1.0 × 10^6^ cells in a 100-mm dish and incubated for 2 days prior to treatment with sodium-β-hydroxybutyrate. Cells were washed twice with 5% mannitol and detached by treatment with trypsin–EDTA. Cell pellets were resuspended in 1 ml methanol and sonicated for 30 s. Cell suspensions were mixed with 1 ml chloroform and 0.4 ml ultra-pure water and vortexed for 30 s. After centrifugation at 2300×*g* at 4 °C for 5 min, the aqueous layers were filtered through an UltrafreeMC-PLHCC (5-kDa cutoff) filter (Human Metabolome Technologies, Yamagata, Japan) at 9100×*g* at 4 °C for 2.5 h to remove proteins and phospholipids. The filtrates were lyophilized and dissolved in 25 μl ultra-pure water. CE-TOF-MS analysis was carried out using an Agilent 7100 CE System equipped with an Agilent 6230 TOF-MS System (Agilent Technologies, CA). Raw data were processed with MassHunter software (Qualitative and Quantitative Analysis, Agilent) for the quantification of metabolites.

### 2.9 Statistical analysis

Values are represented as means ± SEM and tests were performed using GraphPad Prism8 (GraphPad Software Inc., CA). Comparisons between two groups were assessed using unpaired Student's t-test for normally distributed variables. Analysis of multiple comparisons was made using one-way ANOVA followed by Dunnett's or Tukey's post-hoc test. To investigate the relationship between two variables, Pearson's correlation coefficient was used. P values were considered significant at P < 0.05.

## 3 Results

### 3.1 mRNA expression of gluconeogenic genes in kidney increases later than in liver after fasting

**Figure 1:**
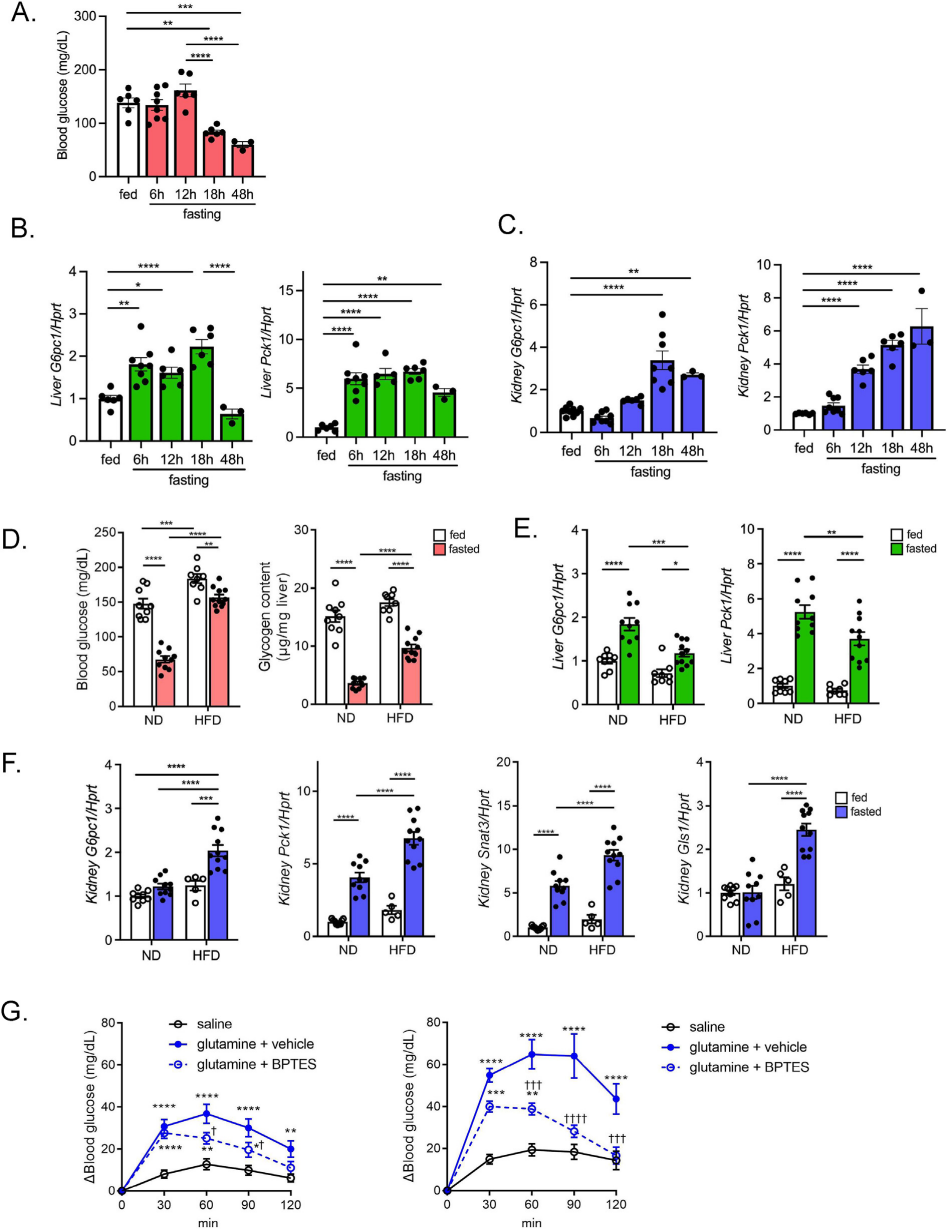
**Gluconeogenic transactivation in liver and kidney after fasting in ND- and HFD-fed mice**. A) Blood glucose in mice in a course of fasting (0, 6, 12, 18, and 48 h). B, C) qPCR analysis of gluconeogenic gene expressions (*G6pc1* and *Pck1*) in liver (B), and kidney (C) in a course of fasting (0, 6, 12, 18, and 48 h) (n = 3 to 8). D) Blood glucose and hepatic glycogen content in ND- and HFD-fed mice in fed and 16-hr fasting state. E, F) qPCR analysis of gluconeogenic gene expression in liver (*G6pc1* and *Pck1*) and kidney (*G6pc1*, *Pck1*, *Snat3*, and *Gls1*) in ND- and HFD-fed mice (n = 8 to 11). G) Glutamine tolerance test in ND-fed (*left*) and HFD-fed mice (*right*). After 16-hr fasting, mice were injected with saline or glutamine. BPTES was orally pre-administered 1 h before glutamine injection. (n = 8, each), G), the asterisk (∗) and the dagger (†) denote a statistically significant difference v.s. saline, and v.s. glutamine + vehicle, respectively. Data are expressed as mean ± SEM. ∗p < 0.05, ∗∗p < 0.01, ∗∗∗p < 0.001, ∗∗∗∗p < 0.0001.

The fasting time-dependent transactivation of the gluconeogenic genes *G6pc1* and *Pck1* were analyzed in liver and kidney in mice. BG levels remained unchanged up to 12-hr fasting but significantly declined at 18 h (Figure 1A). In liver, both *G6pc1* and *Pck1* mRNA expressions began to increase as early as 6-hr fasting and remained high until at least 18 h (Figure 1B). In contrast, the increase in the expressions was delayed in kidney (Figure 1C). While hypoglycemia was induced at 18-hr fasting, no further decline was observed in extended fasting up to 48 h (Figure 1A). After 48-hr fasting, the renal gluconeogenic gene expressions remained high, while hepatic *G6pc1* expression was decreased (Figure 1B,C), suggesting that gluconeogenesis in liver may not play a significant role in BG maintenance under prolonged fasting.

### 3.2 High-fat diet (HFD) feeding potentiates fast-induced expression of gluconeogenic genes in kidney but not in liver

We previously reported that HFD feeding diminishes the fasting-induced decline in glycemia in mice [19], which is reproduced in the present study (Figure 1D). Nevertheless, induction of *G6pc1* and *Pck1* (Figure 1E) and the decrease in glycogen content (Figure 1D) in the liver after fasting were both attenuated in HFD-fed mice, suggesting a contribution of extrahepatic glucose production in the maintenance of glycemia during fasting.

Importantly, expression of the genes involved in renal gluconeogenesis [*G6pc1*, *Pck1,* glutamine transporter (*Snat3*) and glutaminase 1 (*Gls1*)] in the kidney of fasted mice was significantly upregulated by HFD feeding (Figure 1F). To assess the contribution of renal gluconeogenesis on glycemia, we challenged the mice with glutamine, a substrate for renal gluconeogenesis. In ND-fed mice, BG levels were increased after glutamine administration and were significantly reduced by pre-administration of BPTES, a kidney-type glutaminase inhibitor (Figure 1G), implying that renal gluconeogenesis can be quantified by subtracting the BG increase in the absence of BPTES from that in its presence. Furthermore, the increase in BG levels after glutamine loading was significantly larger in HFD-fed mice than that in ND-fed mice, suggesting that HFD feeding lessens the decline in glycemia after fasting through upregulated renal gluconeogenesis.

### 3.3 Ketone body regulation of renal gluconeogenic gene expressions

**Figure 2:**
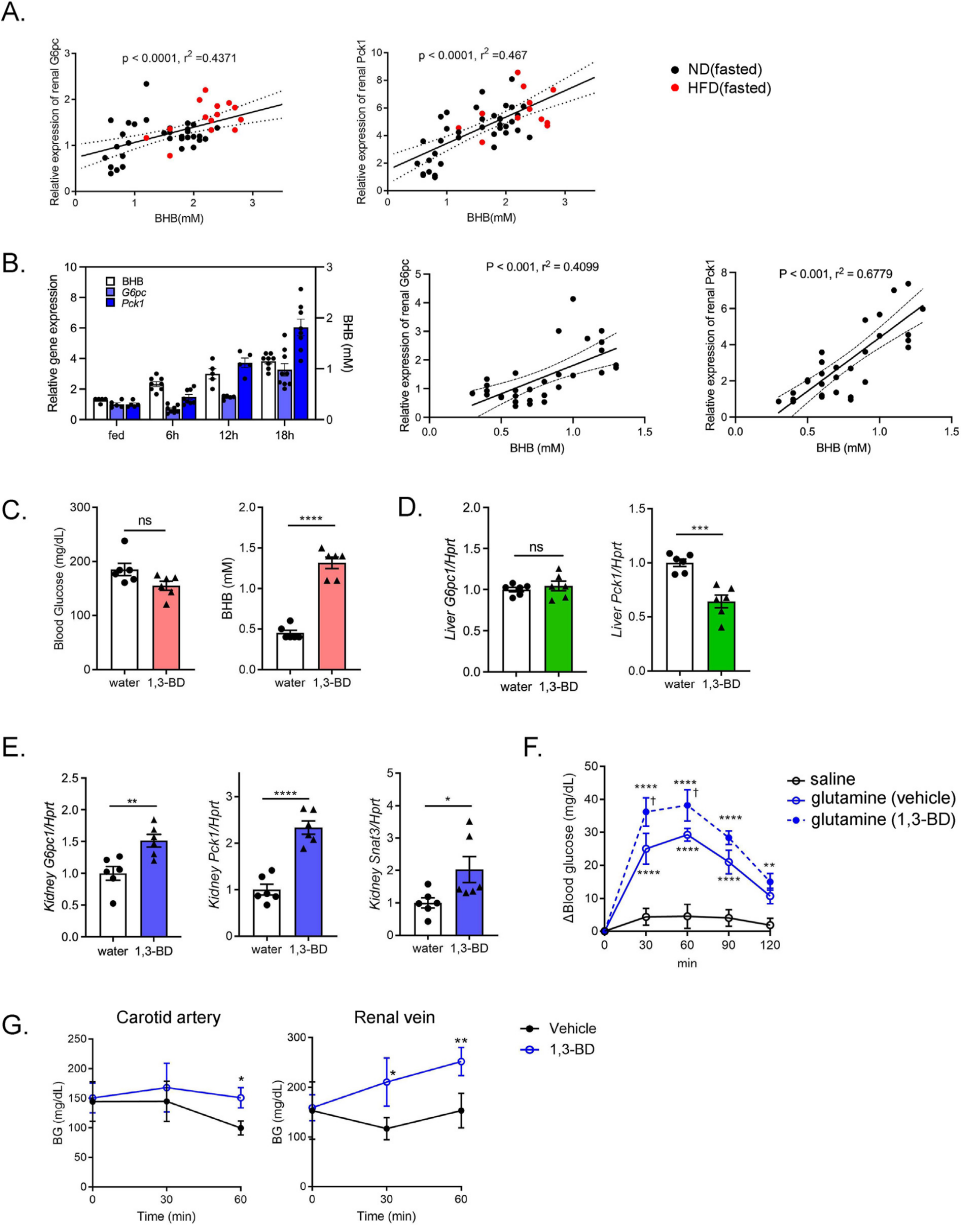
**Ketone body-induced transactivation of renal gluconeogenic gene and glucose release from kidney**. A) Correlation between blood BHB and renal gluconeogenic gene (*G6pc1*, and *Pck1*) expression in fasted ND- and HFD-fed mice. B) Comparison of blood BHB concentration and renal gluconeogenic gene expression at different time points of fasting (0, 6, 12, and 18 h) (n = 5 to 8). Correlations (*left*: BHB vs. renal *G6pc1*, *right*: BHB vs. renal *Pck1*) are shown by scattered plots. C) BG and BHB concentration in mice treated with vehicle (water) or 1,3-BD (n = 6, each). d, e) qPCR analysis of gluconeogenic gene expressions in (D) liver and (E) kidney (*G6pc1* and *Pck1*) in vehicle- or 1,3-BD-treated mice. F) Glutamine tolerance test in vehicle- or 1,3-BD-treated mice (n = 6, each). G) Changes in systemic (*left*: carotid artery) and renal venous BG concentrations (*right*) after vehicle or 1,3-BD treatment (n = 3 to 5), F), the asterisk (∗) and the dagger (†) denote a significant difference v.s. saline, and v.s. glutamine (vehicle), respectively. Data are expressed as mean ± SEM. ∗p < 0.05, ∗∗p < 0.01, ∗∗∗p < 0.001, ∗∗∗∗p < 0.0001.

In the search for the regulator(s) of renal gluconeogenesis, we eventually found a strong positive correlation between renal expression of gluconeogenic genes (*G6pc1* and *Pck1*) and the blood BHB concentrations (Figure 2A). In addition, the time-dependent increase in renal gluconeogenic gene expression after fasting resembled that of blood BHB concentrations, and positive correlations were also observed (Figure 2B). These results suggested that circulating BHB might trigger renal gluconeogenesis.

**Figure 3:**
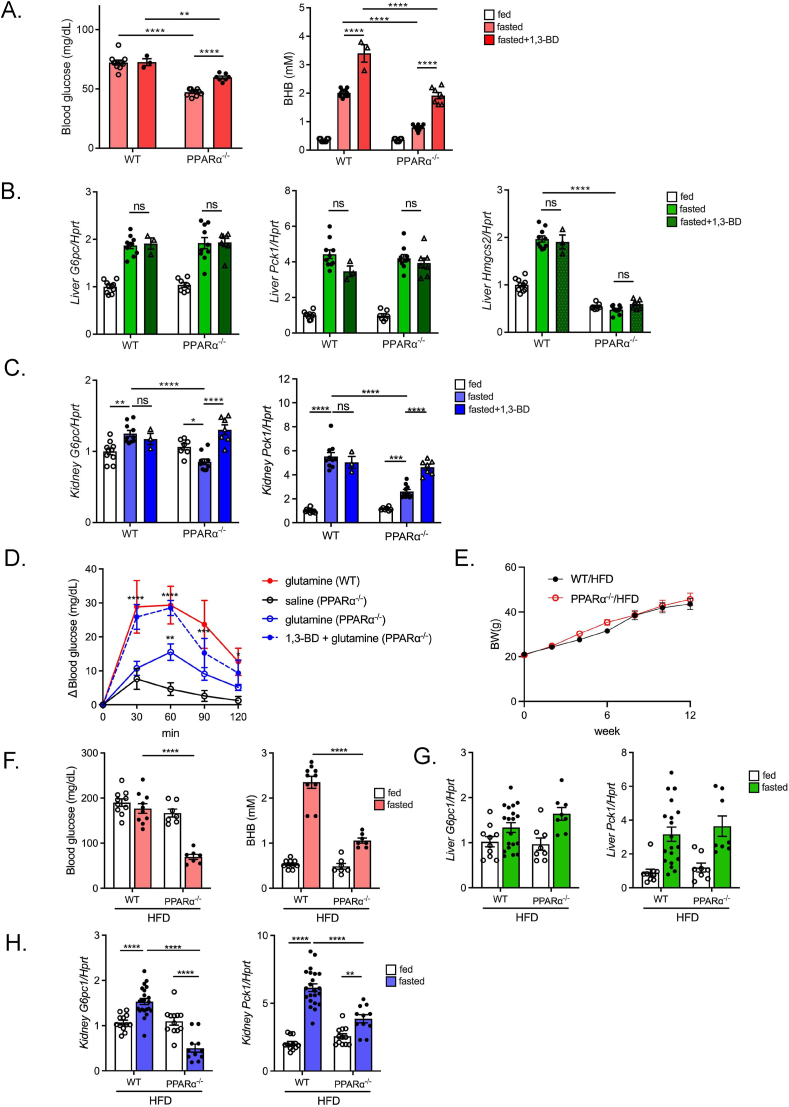
**Downregulation of renal gluconeogenic gene expression and its recovery by 1,3-BD administration in PPARα knockout mice**. A) Blood glucose and BHB concentration in ND-fed WT mice and PPARα^−/−^ mice (fed and 16-hr fasting with/without 1,3-BD treatment) (n = 3 to 10). B, C) qPCR analysis of gluconeogenic (*G6pc1* and *Pck1*) and ketogenic (*Hmgcs2*) gene expression in the liver (B) and kidney (*G6pc1*, *Pck1*, and *Snat3*) (C) in ND-fed WT mice and PPARα^−/−^ mice (fed, and 16-hr fasting with/without 1,3-BD treatment). D) Glutamine tolerance test in 16-hr fasted WT and PPARα^−/−^ mice pretreated with saline or 1,3-BD (n = 4 to 8). E) Alterations in body weight during HFD feeding in WT (WT/HFD) and PPARα^−/−^ (PPARα^−/−^/HFD) mice (n = 5 to 6). F) Blood glucose and BHB concentration in WT/HFD mice and PPARα^−/−^/HFD mice (fed and 16-hr fasting) (n = 7 to 10). G, H) qPCR analysis of gluconeogenic (*G6pc1* and *Pck1*) gene expression in liver (G) and kidney (*G6pc1*, *Pck1*, and *Snat3*) (H) in WT/HFD mice and PPARα^−/−^/HFD mice (fed and 16-hr fasting) (n = 8 to 18). Data are expressed as mean ± SEM. ∗∗p < 0.01, ∗∗∗∗p < 0.0001.

To examine the effect of BHB on renal gluconeogenesis, we administered 1,3-BD, a ketone diester that is preferentially metabolized to BHB *in vivo* by hepatic alcohol dehydrogenase. Although 1,3-BD administration to fed mice did not affect systemic BG levels, it significantly increased the blood BHB concentrations 2 h after its administration to a level comparable to those after 16-hr fasting (Figure 2C). Notably, mRNA expression of *G6pc1* and *Pck1* was significantly increased in the kidney, but not in the liver (Figure 2D,E), suggesting that reduction in hepatic *Pck1* may contribute to the lack of increase in systemic BG after 1,3-BD administration. Pretreatment with 1,3-BD significantly potentiated the glutamine-induced rise in glycemia in fed mice (Figure 2F). In addition, 1,3-BD treatment increased BG levels in the renal vein, but not in the carotid artery (Figure 2G), suggesting that circulating levels of BHB may act as a principal regulator of renal gluconeogenesis. Ketone body in the blood is generated mostly in the liver and the fasting-induced rise in blood ketone body is mediated through hepatic PPARα signaling. Therefore, in order to clarify the physiological role of ketone body on renal expression of gluconeogenic genes, we used PPARα^−/−^ mice that lack fasting-induced ketogenesis [23]. PPARα^−/−^ mice exhibited fasting hypoglycemia (Figure 3A) as previously reported [23]. In PPARα^−/−^ mice, hepatic expression of *Hmgcs2*, a rate-limiting enzyme for ketogenesis, was low and was not transactivated by fasting (Figure 3B). Importantly, 1,3-BD administration ameliorated fasting hypoglycemia in PPARα^−/−^ mice by increasing the blood BHB concentration without transactivation of hepatic *Hmgcs2* expression (Figure 3A,B). In PPARα^−/−^ mice, fasting-induced transactivation of renal *G6pc1* and *Pck1* was attenuated compared with that in WT mice, whereas the transactivation of hepatic *G6pc1* and *Pck1* was maintained similarly to that in WT mice (Figure 3B,C). Intriguingly, renal *G6pc1* and *Pck1* expressions were induced by 1,3-BD administration (Figure 3C). In accord with this observation, the glutamine-induced rise in BG was enhanced in 1,3-BD treated PPARα^−/−^ mice (Figure 3D). These data suggest that renal gluconeogenesis is regulated by circulating BHB.

To investigate the contribution of renal gluconeogenesis to fasting hyperglycemia in diet-induced obese (DIO) mice, PPARα^−/−^ mice were fed HFD. Interestingly, HFD-fed PPARα^−/−^ (PPARα^−/−^/HFD) mice exhibited fasting hypoglycemia despite their obesity being comparable to that of HFD-fed WT (WT/HFD) mice (Figure 3E,F). In fasting, renal gluconeogenic gene expression in PPARα^−/−^/HFD mice was concurrently suppressed compared with that in WT/HFD mice, whereas hepatic gluconeogenic gene expression was maintained (Figure 3G,H), suggesting that BHB-induced renal gluconeogenic gene transactivation substantially contributes to fasting hyperglycemia in WT/HFD mice.

### 3.4 Enhanced BHB production induces renal gluconeogenic gene expression in other animal models

**Figure 4:**
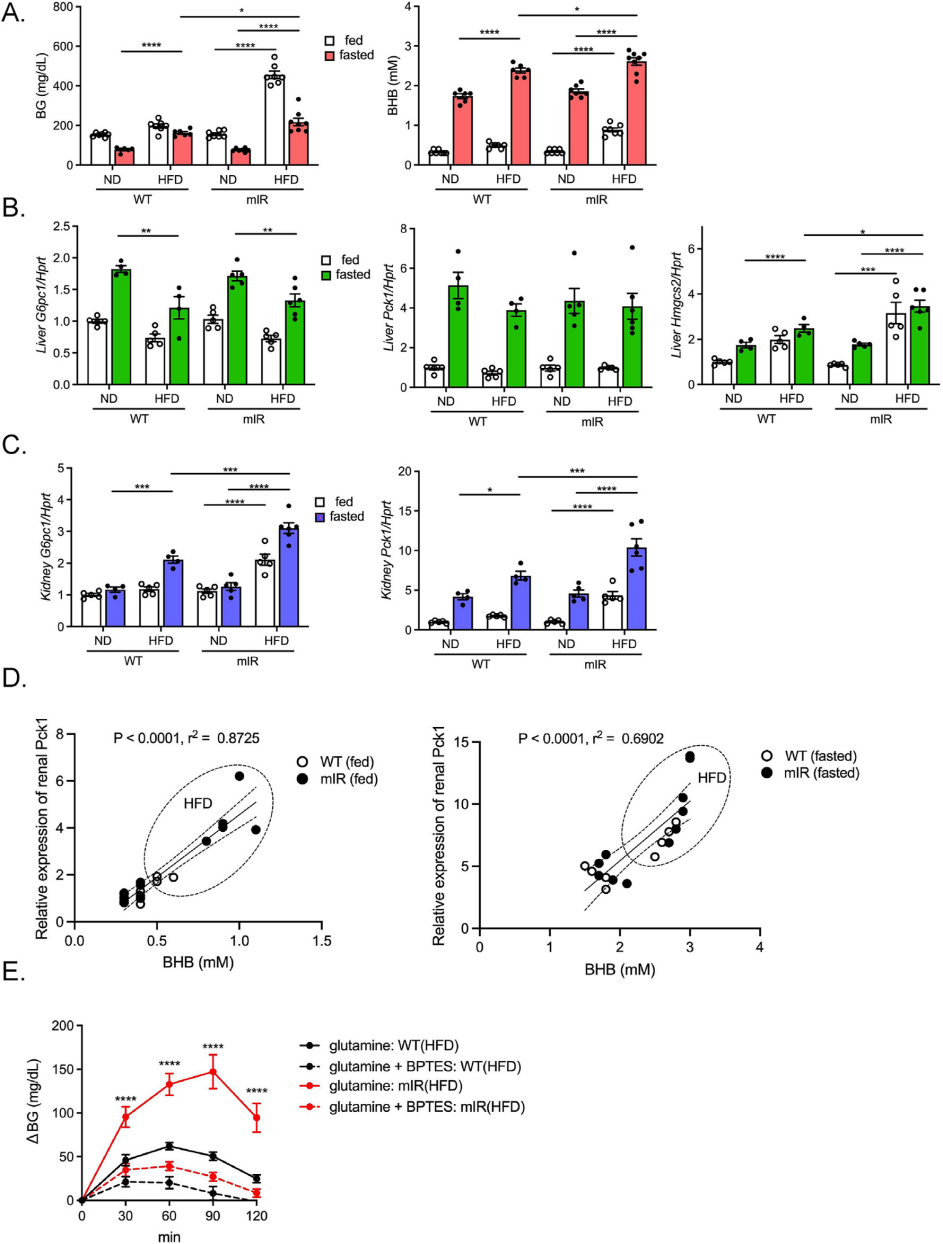

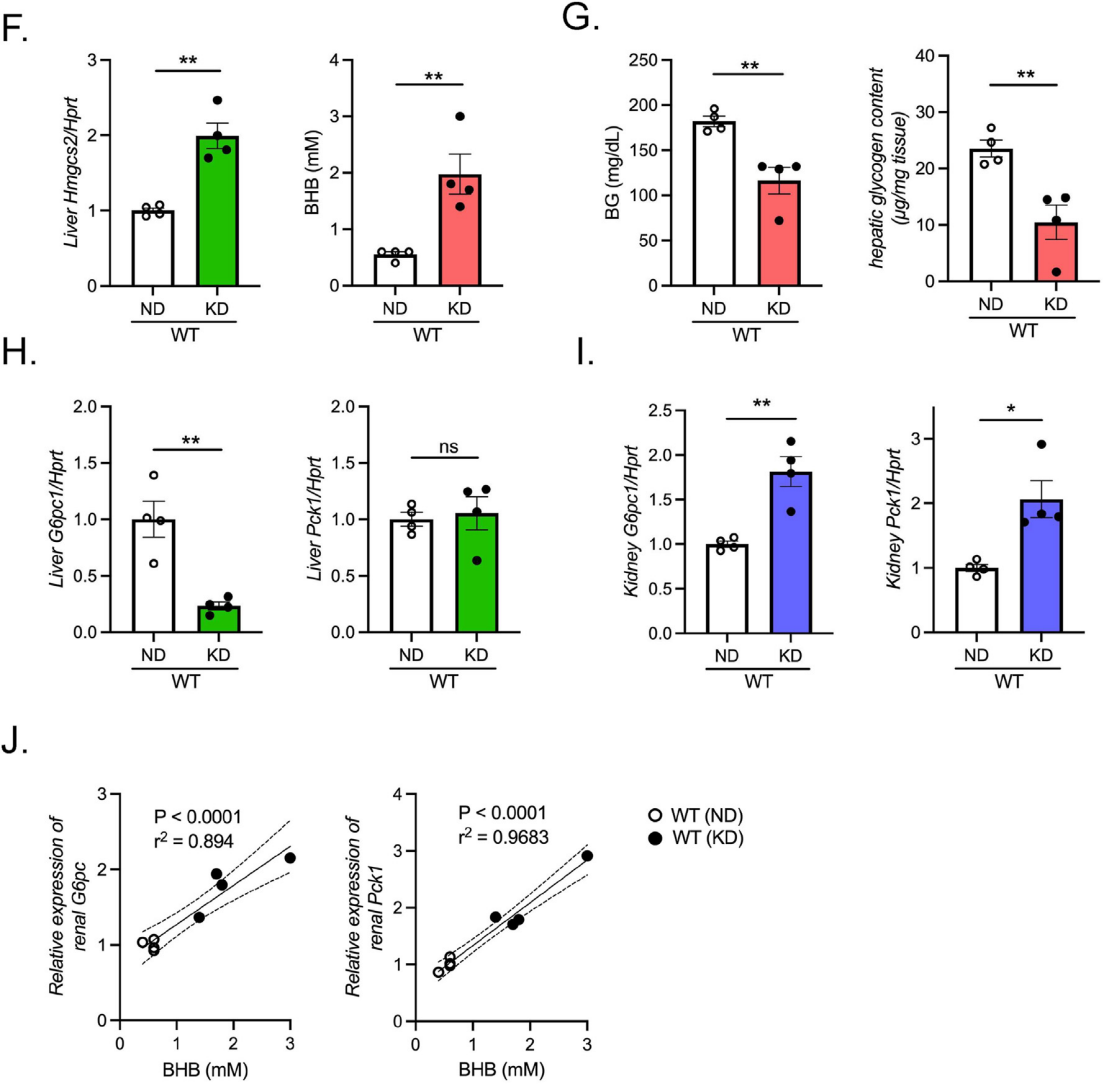
**Enhanced ketogenesis elicits renal gluconeogenic gene transactivation in insulin receptor mutant (mIR) mice and ketogenic diet-fed mice**. A) BG and BHB concentration in ND- and HFD-fed WT and mIR mice (n = 6–8). B) qPCR analysis of gluconeogenic (*G6pc1* and *Pck1*) and ketogenic (*Hmgcs2*) gene expression in liver (B) and kidney (*G6pc1*, *Pck1*, and *Snat3*) (C) in ND- and HFD-fed WT mice and mIR mice (n = 4–6). (D) Correlation between blood BHB and renal *Pck1* expression in WT and mIR mice (*left*; fed state, *right*; fasted state). E) Glutamine tolerance test in 16-hr fasted HFD-fed WT and mIR mice (n = 10, each). BPTES was orally pre-administered 1 h before glutamine injection. F) qPCR analysis of hepatic *Hmgcs2* and blood BHB concentration in ND- and KD-fed mice (n = 4, each). G) Blood glucose level and hepatic glycogen content in ND- and KD-fed mice. H, I) qPCR analysis of gluconeogenic gene (*G6pc1* and *Pck1*) expression in liver (H) and kidney (*G6pc1* and *Pck1*) (I) in ND- and KD-fed mice. J) Correlation of blood BHB and renal *G6pc1* or *Pck1* expression in ND- and KD-fed mice. Data are expressed as mean ± SEM. ∗p < 0.05, ∗∗p < 0.01, ∗∗∗p < 0.001, ∗∗∗∗p < 0.0001.

To clarify the relationship between blood BHB levels and renal gluconeogenesis, we examined other animal models harboring elevated blood BHB. We previously reported that HFD-fed heterozygous mIR (mIR/HFD) mice exhibited an insulin-resistant phenotype [19], showing hyperglycemia even in the fasted state (Figure 4A). Notably, mIR/HFD mice showed a higher blood BHB concentration in both fed and fasted state (Figure 4A). Unlike those in the refed condition after 16-hr fasting, the patterns of hepatic *G6pc1* and *Pck1* expression were similar in WT/HFD and mIR/HFD mice. Notably, the hepatic expression level of *Hmgcs2* was elevated in mIR/HFD mice, possibly due to hepatic insulin resistance. (Figure 4B). On the other hand, renal *G6pc1* and *Pck1* expression in mIR/HFD mice was significantly upregulated compared to that in WT/HFD mice in both fed and fasted state (Figure 4C). Furthermore, a positive correlation between renal *Pck1* expression and blood BHB levels was observed in both fed and fasted states (Figure 4D). Moreover, the glutamine-induced rise in glycemia was markedly enhanced in mIR/HFD mice (Figure 4E). Thus, in mIR/HFD mice, unsuppressed lipolysis in adipocytes as we previously reported, and enhanced hepatic *Hmgcs2* expression due to hepatic insulin resistance may potentiate BHB production and result in renal gluconeogenic gene transactivation.

We then investigated the effect of KD feeding on renal gluconeogenesis. KD feeding would be expected to exert beneficial effects such as body weight reduction and amelioration of BG by shifting the energy source from carbohydrate to fat. In WT mice fed a carbohydrate-free 90% fat KD (WT/KD mice) for 2 weeks, hepatic *Hmgcs2* expression was upregulated, resulting in a marked increase in blood BHB (Figure 4F). In addition, during KD feeding, the mice showed significant reductions in BG levels and hepatic glycogen content (Figure 4G). In the liver, *G6pc1* expression was markedly decreased, but *Pck1* expression was not changed (Figure 4H). In contrast, renal *G6pc1* and *Pck1* expression in WT/KD mice was markedly upregulated (Figure 4I). Notably, a positive correlation between renal gluconeogenic gene expression and the blood BHB concentration was observed in WT/KD mice (Figure 4J). On the other hand, KD-fed PPARα^−/−^ mice (PPARα^−/−^/KD) died after marked hypoglycemia for a few days after KD feeding (data not shown). These findings demonstrate that renal gluconeogenic gene expression is regulated by circulating blood BHB in various physiological and pathophysiological conditions.

### 3.5 BHB upregulates Pck1 protein expression in renal proximal tubules

**Figure 5:**
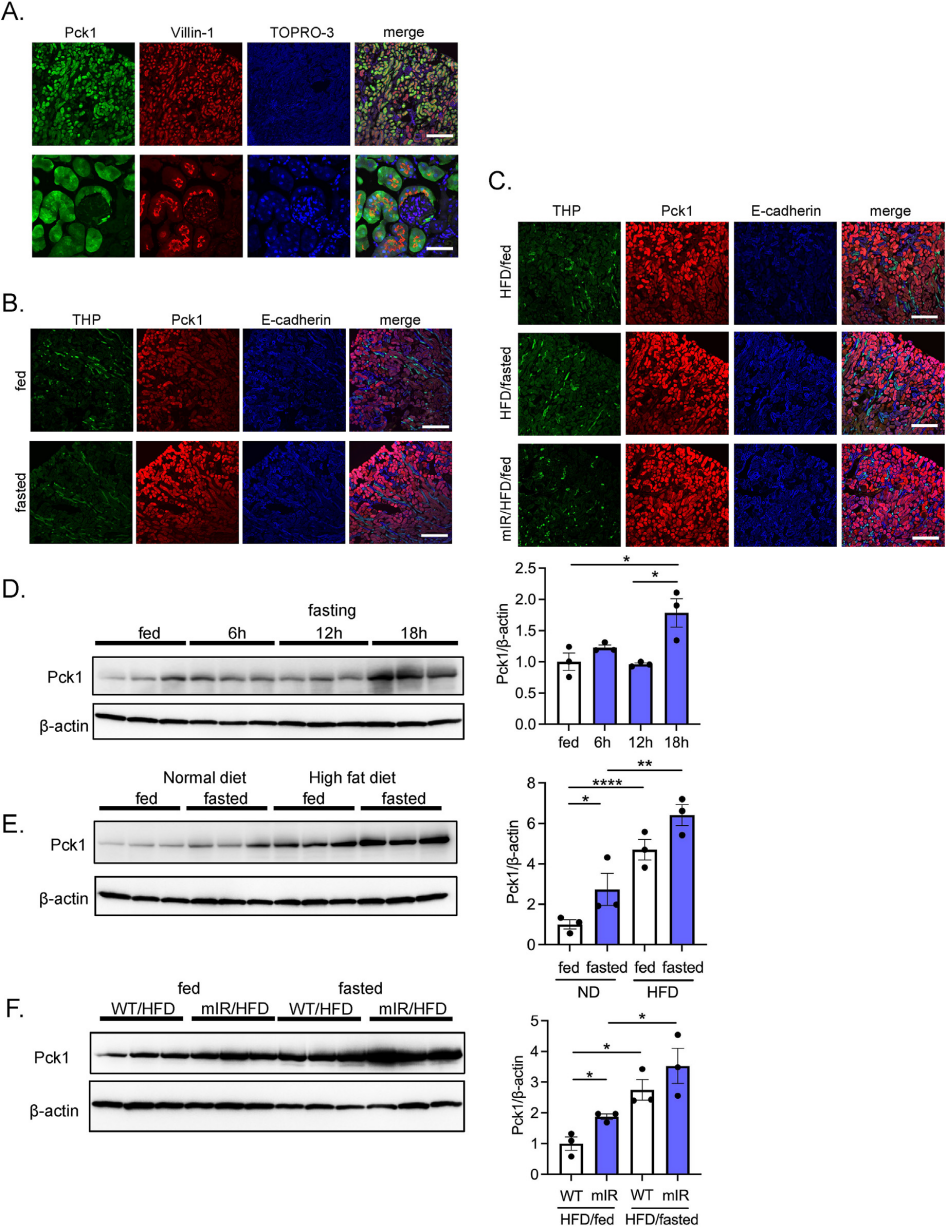

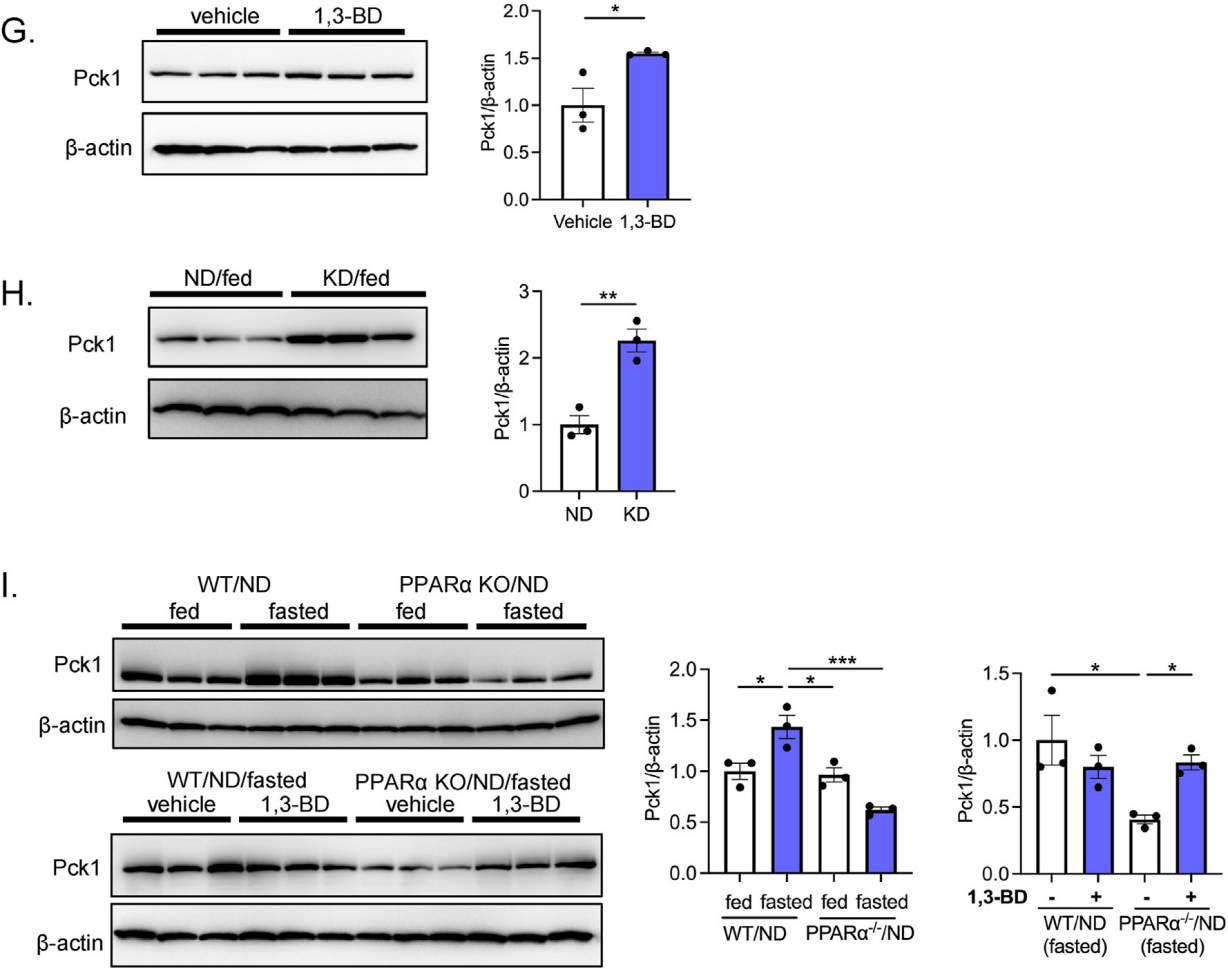
**Renal localization and protein expression levels of PCK1 in various animal models**. A) Determination of renal PCK1 distribution by immunostaining. Renal sections were coimmunostained with PCK1 and villin-1, a marker for PTs. (*upper*: low magnification, *lower*: high magnification). B) Upregulation of PCK1 intensity in 16-hr fasted mouse renal PTs. Coimmunostaining of PCK1 with THP, a marker for thick ascending limb of Henle, and E-cadherin, a marker for distal tubules and collecting duct. C) Upregulation of PCK1 intensity in renal PTs of HFD-fed mice (fed and fasted) and mIR (fed) mice. D) Western blot analysis of the PCK1 protein expression level in renal cortex during fasting. E-I) PCK1 protein expressions were investigated (E) in ND- and HFD-fed mouse renal cortex (fed and 16-hr fasted), (F) in HFD-fed WT and mIR mouse renal cortex (fed and 16-hr fasted), (G) in vehicle or 1,3-BD-treated mouse renal cortex, (H) in ND- and KD-fed mouse renal cortex, and (I) ND-fed WT and PPARα^−/−^ mouse renal cortex (*upper*: fed and 16-hr fasted, *lower*: vehicle and 1,3-BD treatment in 16-hr fasted WT and PPARα^−/−^ mice). Relative PCK1 protein levels are normalized to the total amount of β-actin. Data are expressed as mean ± SEM. ∗p < 0.05, ∗∗p < 0.01, ∗∗∗p < 0.001, ∗∗∗∗p < 0.0001.

To examine the effect of BHB on protein expression of a gluconeogenic gene, we conducted immunofluorescent and western blot analysis for Pck1. First, we determined regional localization of Pck1 in the kidney by immunostaining. Pck1 immunoreactivity was observed exclusively in the PTs by the marker protein villin-1 (Figure 5A). However, Pck1 was not detected in the thick ascending limb of Henle, as assessed by THP immunostaining, or distal tubules or collecting ducts as assessed by E-cadherin staining. Furthermore, Pck1 immunoreactivity was enhanced by 16-hr fasting, indicating that upregulation of Pck1 protein expression is specifically induced in the PTs (Figure 5B). Pck1-positive staining was also augmented in PTs of WT/HFD mice (Figure 5C). Notably, intense Pck1 staining was observed even in the fed state in mIR/HFD mice (Figure 5C).

To quantify the Pck1 protein levels in the kidney, we performed western blot analysis using renal cortex lysate. Significant upregulation of renal Pck1 expression was observed in 18-hr fasted mice, with induction delayed compared with that of *Pck1* gene expression (Figure 1, Figure 5D). Notably, renal Pck1 protein expression was markedly upregulated in WT/HFD mice (Figure 5E). Compared with WT/HFD mice, mIR/HFD mice showed an even higher level of Pck1 expression in both fed and fasted state (Figure 5F). Furthermore, 1,3-BD administration and KD feeding also upregulated Pck1 protein expression (Figure 5G,H). Importantly, fasting-induced Pck1 upregulation was absent in PPARα^−/−^ mice, but 1,3-BD administration significantly increased its expression (Figure 5I). These data strongly suggest that renal Pck1 protein levels are increased in response to BHB-mediated transactivation of *Pck1*.

### 3.6 BHB promotes glucose production via glutamine catabolism in proximal tubule cells

**Figure 6:**
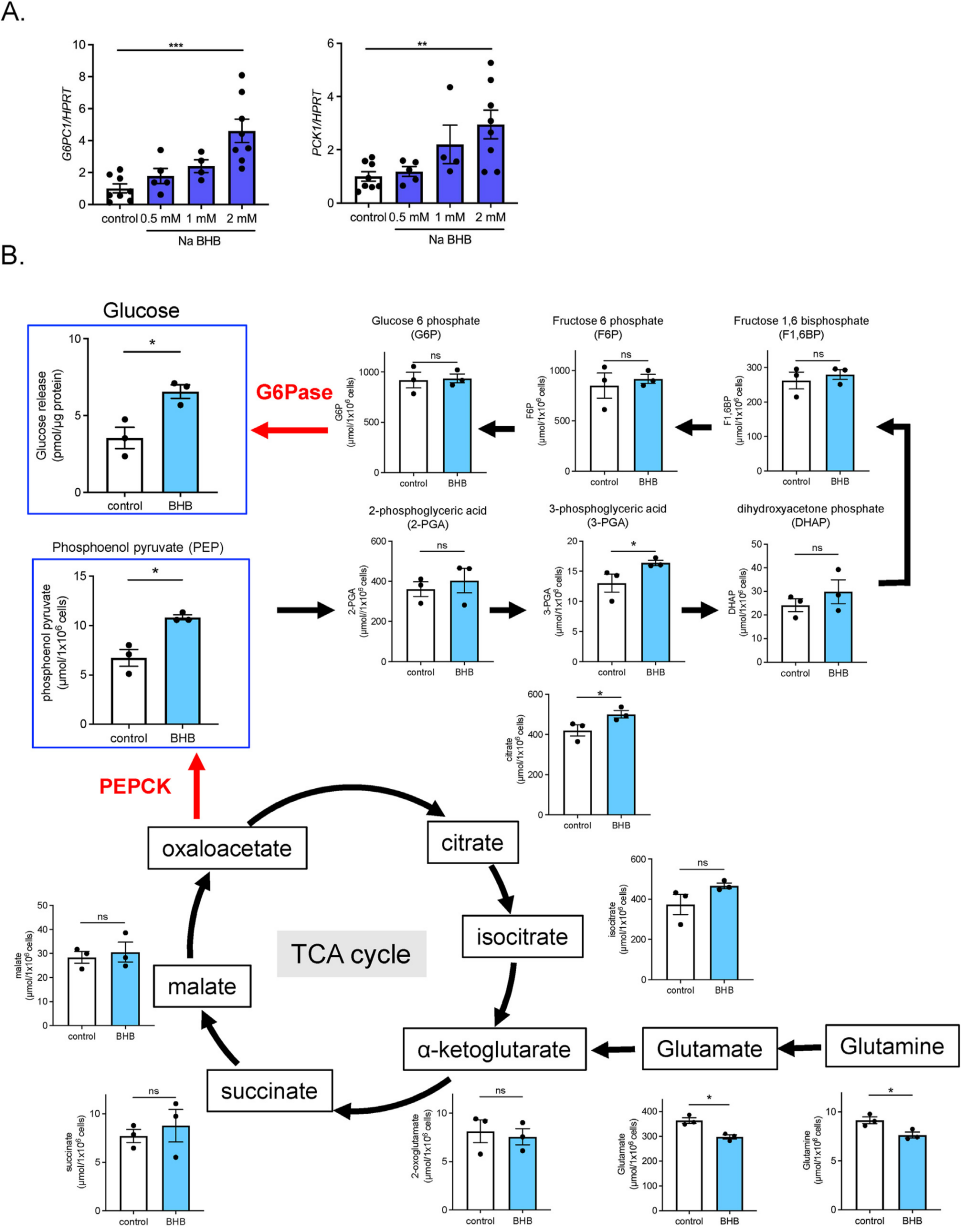
**Metabolomic analysis** of **gluconeogenesis in HK-2 cells**. A) Dose-dependent effects of Na BHB (2 mM) on gluconeogenic gene expression (*G6PC1* and *PCK1*) in HK-2 cells (n = 4 to 8). B) Metabolomic analysis of gluconeogenic and citric acid cycle intermediates in HK-2 cells after Na BHB (2 mM) or vehicle treatment (n = 3, each). Glucose release into medium was measured separately. Data are expressed as mean ± SEM. ∗p < 0.05, ∗∗p < 0.01, ∗∗∗p < 0.001.

To investigate the regulatory mechanism of renal gluconeogenesis by BHB, we examined the direct effect of BHB on renal PTs using HK-2 cells, a human derived immortalized PT cell line. We used Na BHB to avoid acidification of the medium. BHB treatment induced *G6PC* and *PCK1* expression in a dose-dependent manner (Figure 6A). To determine the effect of BHB-mediated transactivation on gluconeogenesis, we conducted metabolome analysis using HK-2 cells (Figure 6B). Interestingly, phosphoenolpyruvate (PEP), an important intermediate of gluconeogenesis, was significantly increased, while glutamine content was decreased by BHB-treatment. Furthermore, BHB-treated HK-2 cells exhibited significantly increased glucose release into medium, indicating that BHB transactivates gluconeogenic gene expression and promotes gluconeogenesis in these cells.

**Figure 7:**
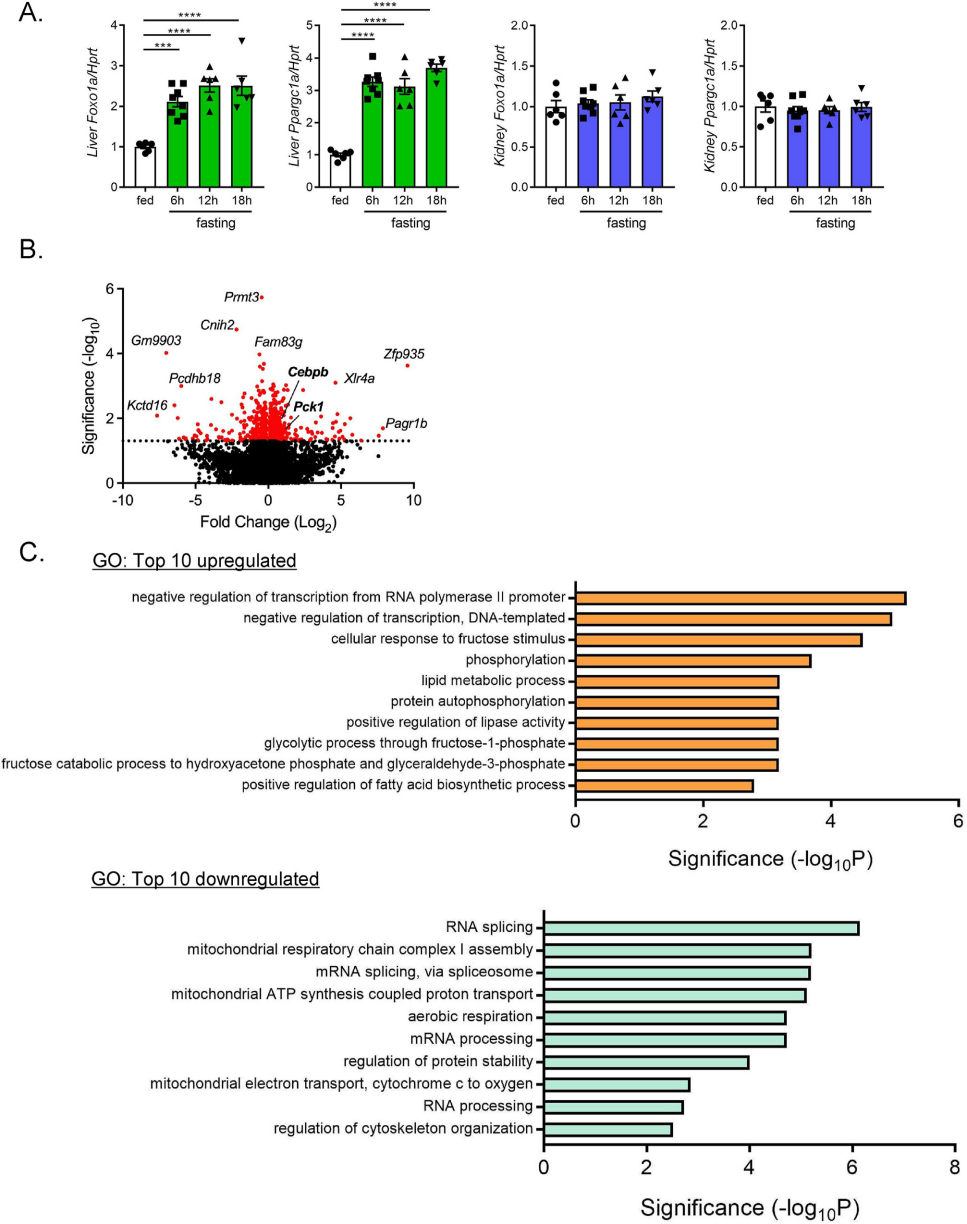

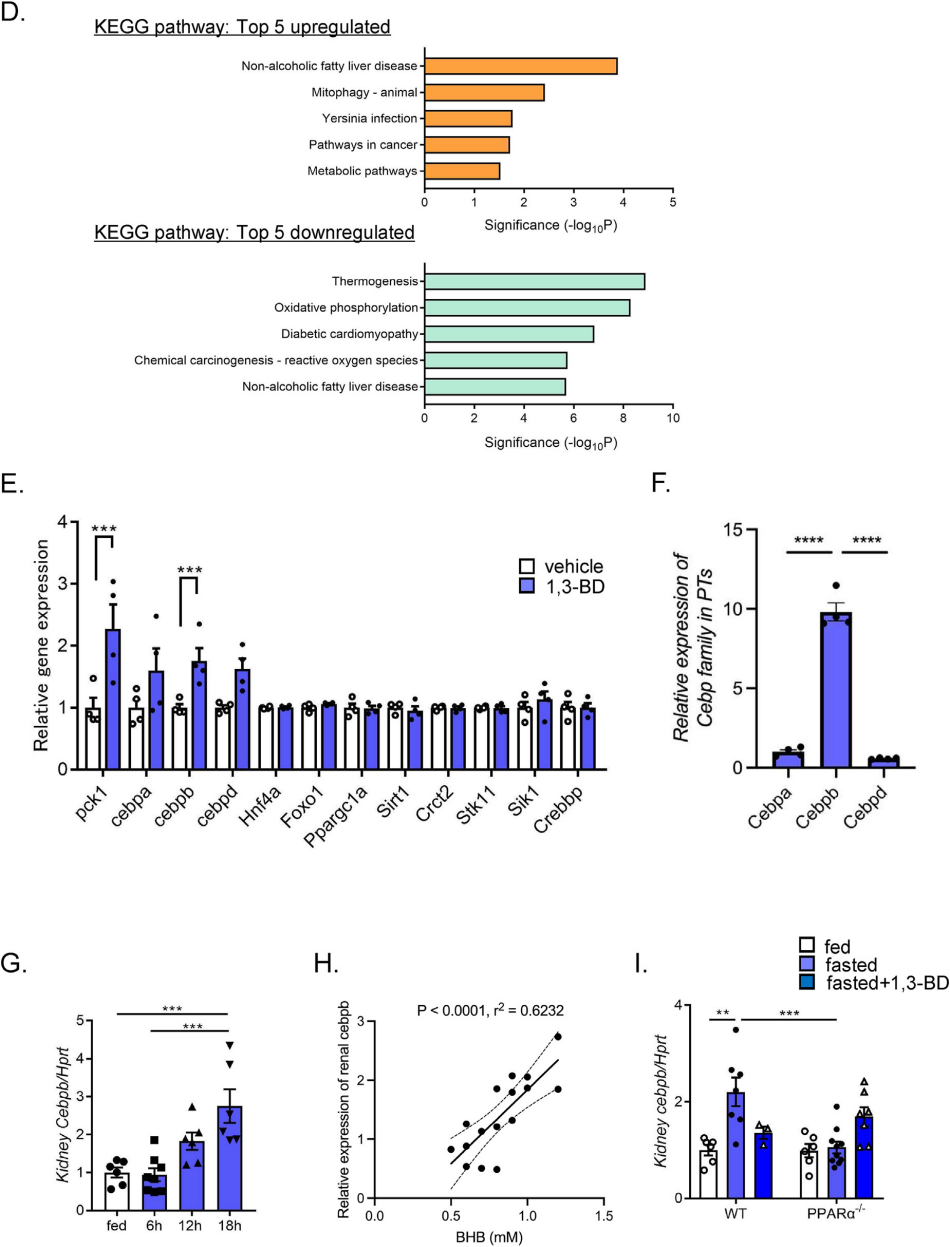
**RNA-seq analysis of isolated PTs from 1,3-BD and vehicle treated mice**. A) qPCR analysis of hepatic and renal *Foxo1a* and *Ppargc1a* expressions in a course of fasting. RNA-seq analysis was performed using isolated PTs from 1,3-BD or vehicle-treated mice (n = 6 to 8). (B) Volcano plot, the results of (C) gene ontology (GO) analysis, and (D) pathway analysis are shown. E) Comparison of expression levels of gluconeogenesis-related genes in isolated PTs in 1,3-BD or vehicle-treated mice (n = 4, each). F) Comparison of the relative transcript amount of *Cebp* family in PTs. Data are based on RNA-seq analysis. G) qPCR analysis of renal *cebpb* expression in a course of fasting (n = 6 to 8), and (H) its correlation with blood BHB. I) qPCR analysis of renal *cebpb* expression in ND-fed WT and PPARα^−/−^ mice (fed, 16-hr fasted and 1,3-BD treated after 16-hr fasting) (n = 3 to 10). Data are expressed as mean ± SEM. ∗∗p < 0.01, ∗∗∗p < 0.001, ∗∗∗∗p < 0.0001.

To further investigate the molecular mechanism of BHB-induced gluconeogenic gene transactivation, we examined hepatic and renal expression levels of transcriptional factors that are involved in gluconeogenesis (Figure 7A). In liver, PGC-1α (*Ppargc1*) and FoxO1(*Foxo1*), the principal transcriptional regulators of hepatic gluconeogenesis, were markedly upregulated by fasting. However, they were unaltered in kidney, clearly indicating that hepatic and renal gluconeogenesis are regulated through distinct transcriptional regulatory mechanisms. To identify the key transcriptional regulators in the kidney, we performed RNA-seq analysis using PTs isolated from mice treated with 1,3-BD or vehicle. Totally, 587 genes (328 upregulated, 259 downregulated) that were changed significantly by 1,3-BD treatment were identified (Figure 7B). GO analysis showed that many of the transcriptional regulators were upregulated by 1,3-BD treatment (Figure 7C). On the other hand, gene expression associated with RNA splicing or mRNA processing were downregulated. Pathway analysis showed upregulation of metabolism-associated gene expression including *Pck1* (Figure 7D). However, major transcriptional regulators for hepatic gluconeogenesis including *Ppargc1, Foxo1, Hnf4a* were not changed (Figure 7E). Intriguingly, genes of the C/EBP family including *Cebpa*, *Cebpb*, and *Cebpd* were upregulated by 1,3-BD treatment. Among them, *Cebpb* showed the highest expression level in PTs (Figure 7F). Importantly, *Cebpb* expression was proportionally increased with fasting-time and renal gluconeogenic gene expression (Figure 7G,H). In PPARα ^−/−^ mice, *Cebpb* expression was downregulated in the fasting state, but was rescued by 1,3-BD administration (Figure 7I). These findings suggest that C/EBPβ plays a key role in BHB-regulation of renal gluconeogenesis.

**Figure 8:**
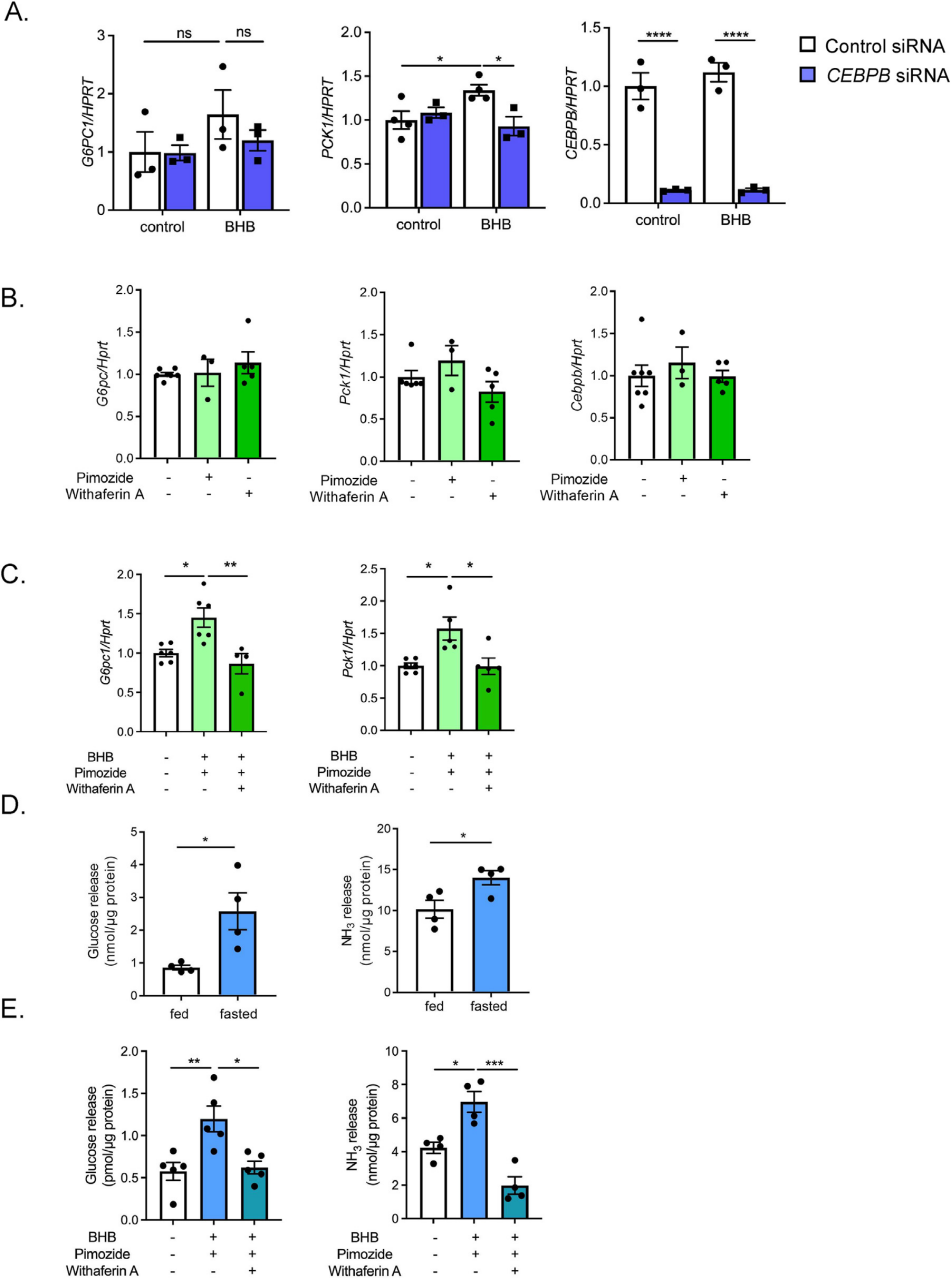
Role of C/EBPβ in regulation of renal gluconeogenesis by BHB. A) Effect of *CEBPB* siRNA transfection on BHB-mediated gluconeogenic gene expression (*G6PC1*, *PCK1* and *CEBPB*) in HK-2 cells. Cells were treated with or without BHB at 72-hr after siRNA transfection (n = 3 to 4). B) Effect of single treatment of inhibitors (pimozide, a SCOT inhibitor, and withaferin A (a C/EBPβ inhibitor) on gluconeogenic genes (*Pck1* and *G6pc1*) and *Cebpb* in isolated PTs (n = 4 to 6). C) Inhibitory effects of withaferin A on BHB-mediated gluconeogenic gene (*G6pc1* and *Pck1*) expression. D) Glucose and NH_3_ release from isolated PTs from fed- and 16hr fasted-mice. E) Glucose and NH_3_ release from isolated PTs after vehicle or BHB treatment (n = 4 to 6). Data are expressed as mean ± SEM. ∗p < 0.05, ∗∗p < 0.01, ∗∗∗∗p < 0.0001.

We then investigated the role of C/EBPβ on BHB-elicited renal gluconeogenesis. BHB treatment of HK-2 cells significantly induced *PCK1* mRNA expression together with a tendency to increase *CEBPB* mRNA expression. Knockdown of *CEBPB* by siRNA transfection suppressed BHB-mediated *PCK1* upregulation, suggesting that CEBPB is involved in BHB-mediated *PCK1* transactivation (Figure 8A). The tendency of BHB-induced *G6PC1* to increase was also inhibited in CEBPB siRNA-treated HK-2 cells. We also examined the effects of BHB on PTs isolated from WT mice. These PTs were treated with Na BHB in combination with pimozide, a SCOT inhibitor, to suppress consumption of BHB [24] and/or withaferin A, a compound with pleiotropic effects including C/EBPβ inhibition [25,26]. Neither pimozide nor withaferin A treatment altered basal expressions of the gluconeogenic genes in PTs (Figure 8B). However, BHB treatment enhanced gluconeogenic gene transactivation in PTs, which in turn was significantly suppressed by withaferin A (Figure 8C). Furthermore, SCOT inhibitor did not suppress the increase in gluconeogenic gene expression by BHB, implying that utilization of BHB as an energy source may not be required for transactivation of gluconeogenic genes. In addition, we examined glucose and NH_3_ release from isolated PTs. PTs from 16-hr fasted mice showed significantly higher production of glucose and NH_3_ *in vitro* (Figure 8D). Furthermore, BHB treatment enhanced glucose and NH_3_ production that had been suppressed by withaferin A (Figure 8E). Moreover, these responses were retained in PPARα^−/−^ PTs (Fig. S1), suggesting that PPARα signaling in PTs is not required for the gluconeogenic responses. These results indicate that C/EBPβ is an essential transcriptional regulator of BHB-induced upregulation of the renal gluconeogenic genes.

## 4 Discussion

In this study, we show that the blood BHB concentration is positively correlated with renal gluconeogenic gene expression. Furthermore, upregulated hepatic ketogenesis and concurrent upregulation of renal gluconeogenic gene expression are associated with fasting hyperglycemia in DIO mice, an effect that is blunted in PPARα^−/−^ mice. We conclude that renal gluconeogenesis is regulated by the BHB generated in liver.

In the initial fasting state, hepatocytes generate glucose through glycogenolysis and gluconeogenesis. As fasting progresses, glycogen stores are depleted and ketogenesis is induced to supply ketone bodies which serve directly as an alternative energy source. On the other hand, during prolonged fasting, hepatic gluconeogenic gene expression is downregulated while renal gluconeogenesis is maintained. Thus, hepatic and renal gluconeogenic gene expressions are induced by distinct regulatory mechanisms. In fact, unlike the case in hepatic gluconeogenesis, transcriptional upregulation of *FoxO1* and *Ppargc1a* expression is shown in the present study not to be involved in BHB-dependent renal gluconeogenic gene transactivation, indicating that this ketone body is the primary mediator of hepato–renal interaction in inducing renal gluconeogenesis in fasting conditions. Moreover, recent evidence indicates that BHB can act as chemical mediators *in vivo* in addition to their role as a direct energy source. For example, BHB directly exerts physiological effects through G protein coupled receptor-mediated signaling or through histone modulation [such as histone deacetylase (HDAC) inhibition or β-hydroxybutyrylation] [27]. Our *in vitro* experiments in HK-2 cells using a SCOT inhibitor showed that utilization of BHB as an energy source is unlikely to participate in renal gluconeogenic gene transactivation**,** suggesting a possible role of ketone body as a humoral signaling molecule. Shimazu et al. reported that BHB infusion upregulates antioxidant gene expression via HDAC inhibition in mouse kidney [28], which suggests circulating BHB regulation of renal gene expression. Even so, we found that the physiological concentration of BHB resulted in only limited HDAC inhibition in HK-2 cells (Fig. S2). On the other hand, increased histone β-hydroxybutyrylation in the kidney has been reported in several ketogenic conditions, including fasting and KD feeding [29,30]. As approximately 80 % of glomerular-filtered BHB is reabsorbed by proximal convoluted tubules [31], the proximal tubules are exposed to excessive BHB under ketogenic conditions and are thereby susceptible to the changes in blood BHB levels.

Zhang et al. [32] reported that BHB regulates glycogenesis in CD8^+^ memory T cells through direct epigenetic modification of Lys 9 of histone H3 (H3K9) in the promoter regions of *Foxo1* and *Ppargc1a,* which resulted in upregulation *Pck1* expression. Contrary to these reports, transactivation of *Foxo1* and *Ppargc1a* was found in this study not to occur in proximal tubules, although histone β-hydroxybutylyration in proportion to blood BHB levels was increased (Fig. S3), suggesting that renal gluconeogenesis may be regulated by histone β-hydroxybutylyration through a mechanism different from that in CD8^+^ memory T cells. We also found by RNA-seq analysis that 1,3-BD treatment transactivated *Cebpb* expression concurrently with *Pck1* upregulation in proximal tubules. Croniger et al. previously reported that *Cebpb* knockout mice show fasting hypoglycemia [33]. Furthermore, C/EBPβ regulates *Pck1* expression through activation of the cAMP response in liver and kidney [34,35]. Thus, C/EBPβ plays a key role in BHB-mediated renal gluconeogenic gene transactivation. On the other hand, *in vitro* experiments showed that BHB directly transactivated SNAT3 but not GLS1 (Fig. S4). However, knockdown or inhibition of C/EBPβ did not affect the BHB-dependent SNAT3 expression, suggesting that other regulatory mechanisms are involved. Further investigation is required to clarify the molecular mechanisms.

Proximal tubules play an important role in reabsorption of nutrition filtered by glomeruli; ∼99 % of filtered glucose is reabsorbed in this segment via Na^+^-dependent glucose transporter (SGLT) 1 and 2, although glucose itself is not utilized as an energy source by proximal tubules due to the lack of glycolytic enzymes. Instead, proximal tubules metabolize fatty acids for their energy production and can generate glucose from non-carbohydrate substrates including lactate, pyruvate, glycerol, and glutamine for other uses. Since glutamine is the main source of renal NH_3_ production, acid-base balance regulation and gluconeogenesis is thought to be coupled in the organ, suggesting the possibility that glycogen storage disease type Ia (GSDIa), attributing to mutation of G6Pase, causes metabolic acidosis in human [36,37]. Furthermore, Verissimo et al. recently reported that renal PCK1 plays essential roles in body acid-base balance regulation through ammonia and bicarbonate synthesis [38]. Considering these findings together, glucose production through renal gluconeogenesis may well serve as a cataplerotic pathway for intermediate metabolites after the generation of ammonia and bicarbonate. In fact, accumulated glucose-6-phoshate (G6P) is aberrantly converted to glycogen and impairs tubular function in *G6pc1* knockout mice [39].

On the other hand, patients with fatty acid oxidation disorder (FAOD) also show fasting hypoglycemia, hypoketonemia, and metabolic acidosis. In FAOD patients, fatty acid β-oxidation is impaired due to genetic mutation in the metabolizing enzymes or fatty acid transporters [40]. The phenotype of fasted PPARα^−/−^ mice resembles that of FAOD patients. In addition, patients having mitochondrial HMG-CoA synthase 2 (HMGCS2) deficiency, a rare autosomal recessive disorder of ketogenesis attributed to mutation of *HMGCS2*, also show fasting hypoglycemia and metabolic acidosis [41]. This evidence supports the physiological importance of BHB-mediated renal gluconeogenesis in acid-base regulation.

Renal gluconeogenesis is known to be coupled with the pathway required for offsetting blood acidification through generation of NH_3_ and HCO_3_^−^ by glutamine catabolism. In this context, the production of glucose in kidney might well be a by-product of a scavenging process involved in acid-base balance regulation. Thus, increased BHB production due to insulin resistance and/or excessive consumption of dietary fat could result in enhanced *de novo* glucose production in the kidney. Similarly, KD feeding induces hyperketonemia and resultant enhanced *de novo* glucose production in the kidney, however, the BG level remains somewhat decreased in mice. We suggest that while lack of carbohydrate in KD (0 % kcal) results in a massive decline in the glucose supply into systemic circulation [29,42], it does not result in hyperglycemia due to increased renal gluconeogenesis. The lack of hyperglycemia by KD in mice accords with the effectiveness of low carbohydrate diet or KD in lowering glycemic levels in diabetes patients [43]. However, our present data on glucose lowering effect of KD in mice is not sufficient to support the safety and clinical benefit of KD in the treatment of diabetes mellitus in human.

## 5 Conclusion

We describe here a novel mechanism whereby blood BHB generated by hepatic ketogenesis under fasting conditions transactivates rate-limiting gluconeogenic gene expression in the kidney to maintain both acid-base balance and *de novo* glucose production. This indirect mechanism illustrates a physiological function of BHB other than direct fuel for the maintenance of BG homeostasis as well as another example of hepato/renal reciprocity.

## Funding

This research was supported by Young Researcher Grants (2020) from the 10.13039/501100008644Japan Diabetes Society (R.H.), 10.13039/501100007664Manpei Suzuki Diabetes Foundation (2022) (R.H.), and 10.13039/501100001691Japan Society for the Promotion of Science KAKENHI (grant numbers: JP23K06343 for R.H. and JP22H02800 for T.M.).

## CRediT authorship contribution statement

**Ryo Hatano:** Writing – review & editing, Writing – original draft, Visualization, Validation, Methodology, Investigation, Funding acquisition, Formal analysis, Data curation, Conceptualization. **Eunyoung Lee:** Validation, Investigation, Data curation. **Hiromi Sato:** Validation, Methodology, Investigation, Data curation. **Masahiro Kiuchi:** Resources, Methodology, Investigation, Data curation. **Kiyoshi Hirahara:** Resources, Methodology, Data curation. **Yoshimi Nakagawa:** Resources, Methodology. **Hitoshi Shimano:** Writing – review & editing, Supervision, Resources. **Toshinori Nakayama:** Writing – review & editing, Supervision, Resources. **Tomoaki Tanaka:** Writing – review & editing, Supervision, Resources. **Takashi Miki:** Writing – review & editing, Writing – original draft, Supervision, Resources, Project administration, Funding acquisition, Conceptualization.

## Declaration of competing interest

The authors declare that they have no known competing financial interests or personal relationships that could have appeared to influence the work reported in this paper.

## Data Availability

Data will be made available on request.

## Abbreviations

1,3-BD: 1,3-butandiol
BPTES: N,N'-[thiobis (2,1-ethanediyl-1,3,4-thiadiazole-5,2-diyl)] bis-benzeneacetamide
C/EBP: CCAAT/enhancer binding protein
FAOD: fatty acid oxidation disorder
Gls1: glutaminase 1
GSD: glycogen storage disease
mIR: insulin receptor *P1195L* mutant
PTs: proximal tubules
SGLT: sodium dependent glucose transporter
Snat3: sodium-coupled neutral amino acid transporter 3
THP: Tamm Horsfall protein

## Footnotes

Appendix A Supplementary data to this article can be found online at https://doi.org/10.1016/j.molmet.2024.101934.
