# Supplementary material for: Hepatic ketone body regulation of renal gluconeogenesis
Source: Mol Metab. 2024 Apr 9;84:101934. doi: 10.1016/j.molmet.2024.101934 (PMC11039402; doi:10.1016/j.molmet.2024.101934)
Supplement: Multimedia component 1 [file mmc1.pptx]

## Slide 1
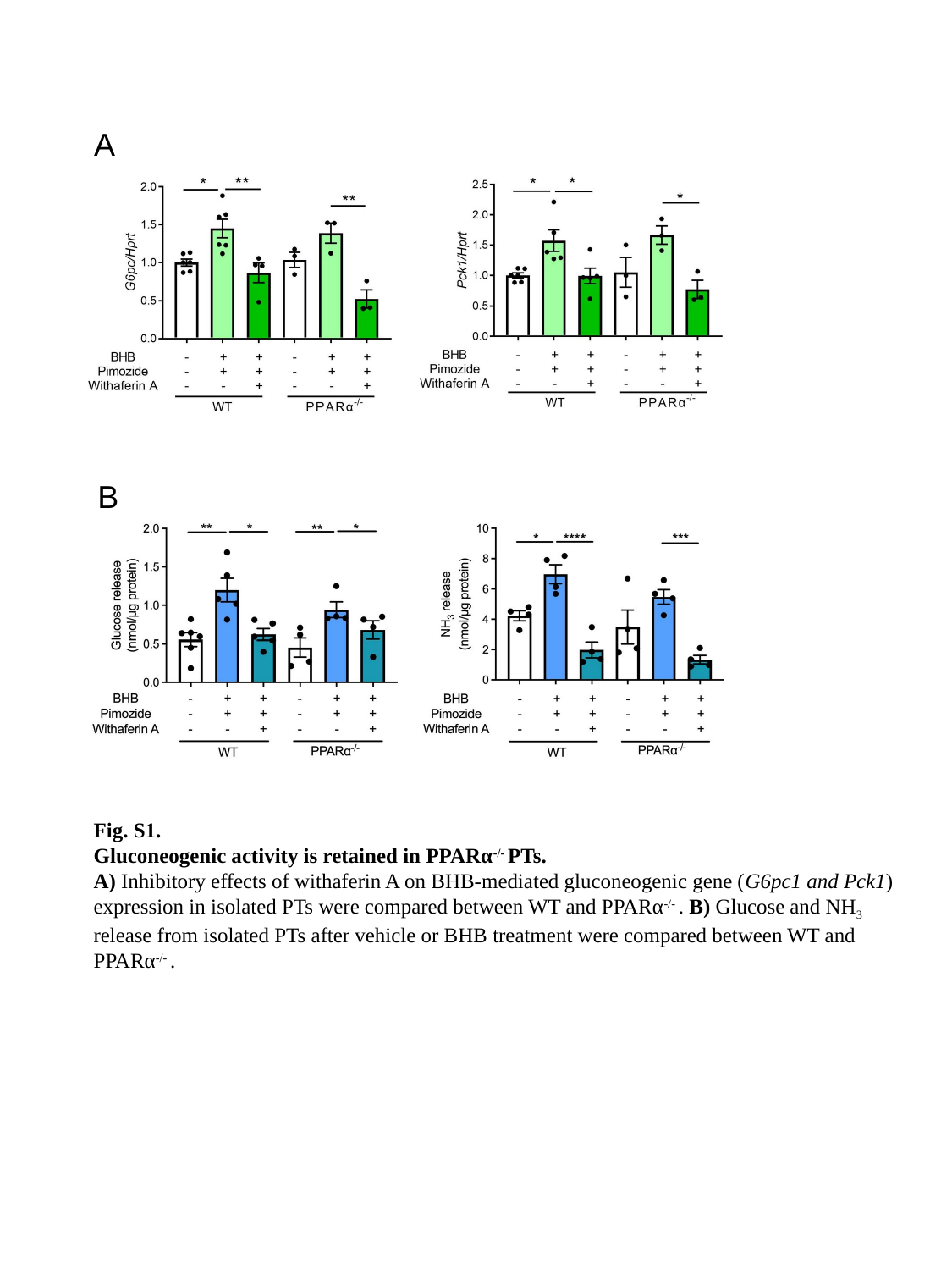

A
B
Fig. S1.
Gluconeogenic activity is retained in PPARα-/- PTs.
A) Inhibitory effects of withaferin A on BHB-mediated gluconeogenic gene (G6pc1 and Pck1) expression in isolated PTs were compared between WT and PPARα-/- . B) Glucose and NH3 release from isolated PTs after vehicle or BHB treatment were compared between WT and PPARα-/- .

## Slide 2
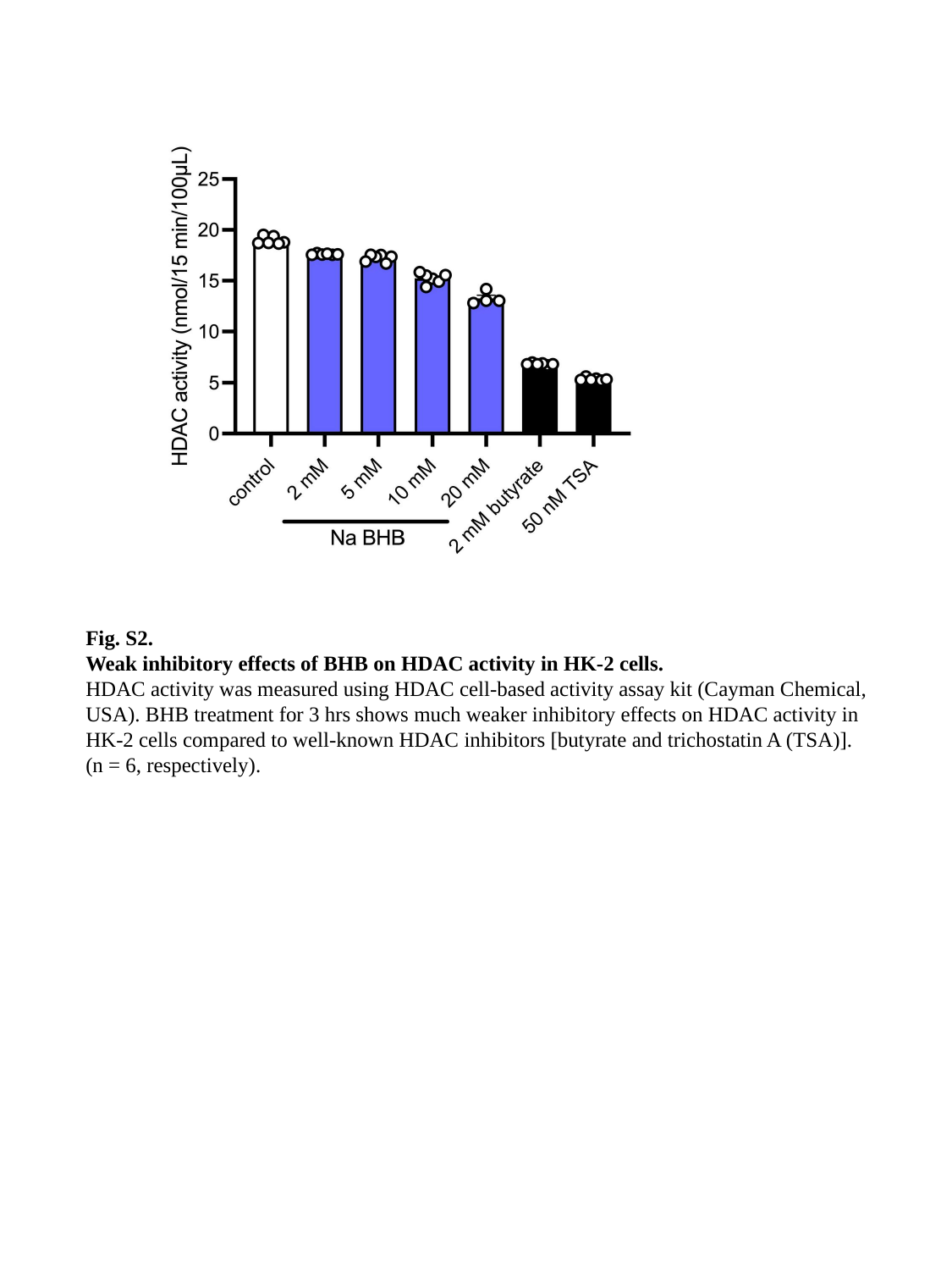

Fig. S2.
Weak inhibitory effects of BHB on HDAC activity in HK-2 cells.
HDAC activity was measured using HDAC cell-based activity assay kit (Cayman Chemical, USA). BHB treatment for 3 hrs shows much weaker inhibitory effects on HDAC activity in HK-2 cells compared to well-known HDAC inhibitors [butyrate and trichostatin A (TSA)].
(n = 6, respectively).

## Slide 3
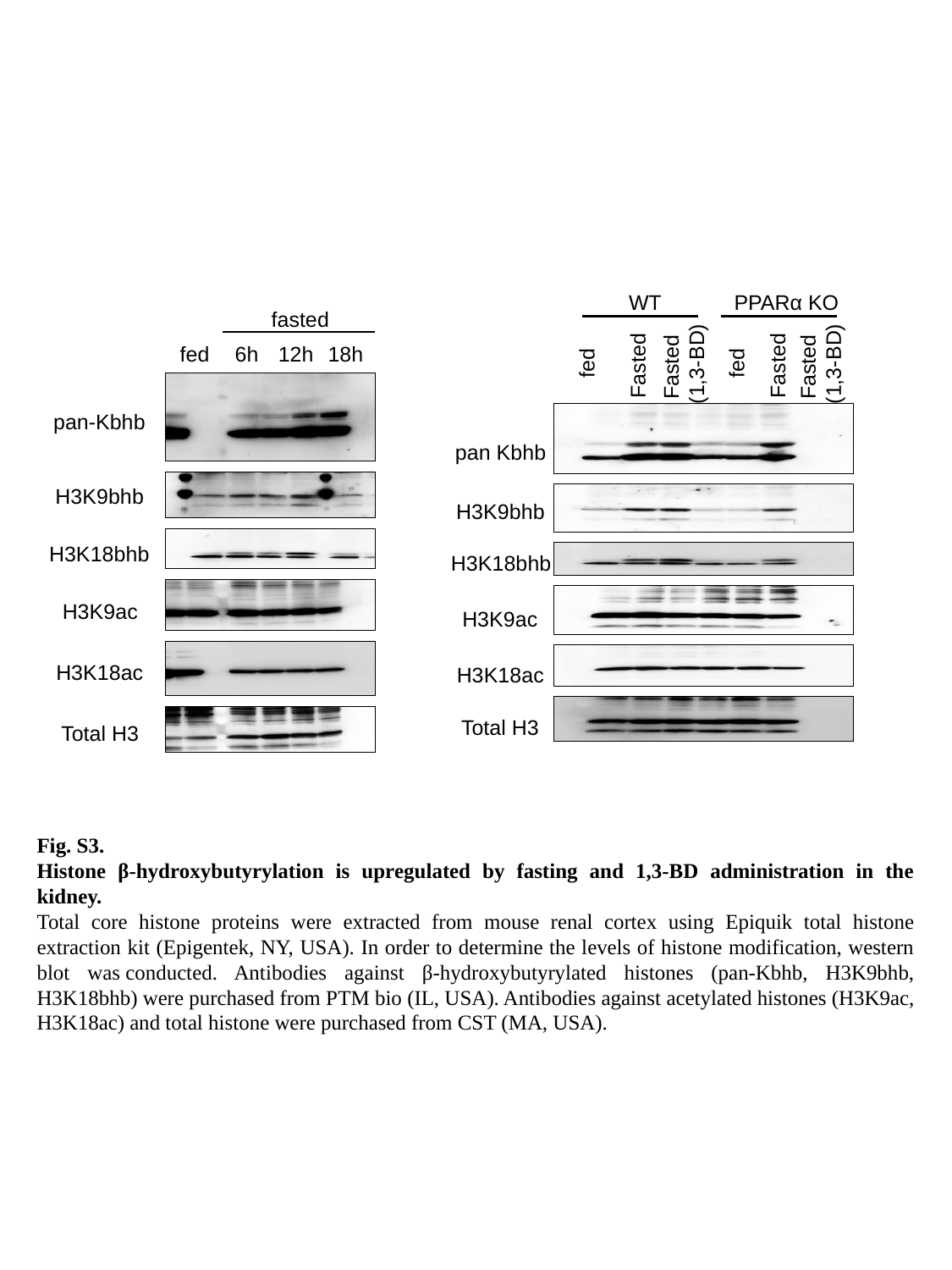

PPARα KO
WT
Fasted
(1,3-BD)
Fasted
(1,3-BD)
fed
fed
Fasted
Fasted
pan Kbhb
H3K9bhb
H3K18bhb
H3K9ac
H3K18ac
Total H3
fasted
fed
6h
12h
18h
pan-Kbhb
H3K9bhb
H3K18bhb
H3K9ac
H3K18ac
Total H3
Fig. S3.
Histone β-hydroxybutyrylation is upregulated by fasting and 1,3-BD administration in the kidney.
Total core histone proteins were extracted from mouse renal cortex using Epiquik total histone extraction kit (Epigentek, NY, USA). In order to determine the levels of histone modification, western blot was conducted. Antibodies against β-hydroxybutyrylated histones (pan-Kbhb, H3K9bhb, H3K18bhb) were purchased from PTM bio (IL, USA). Antibodies against acetylated histones (H3K9ac, H3K18ac) and total histone were purchased from CST (MA, USA).

## Slide 4
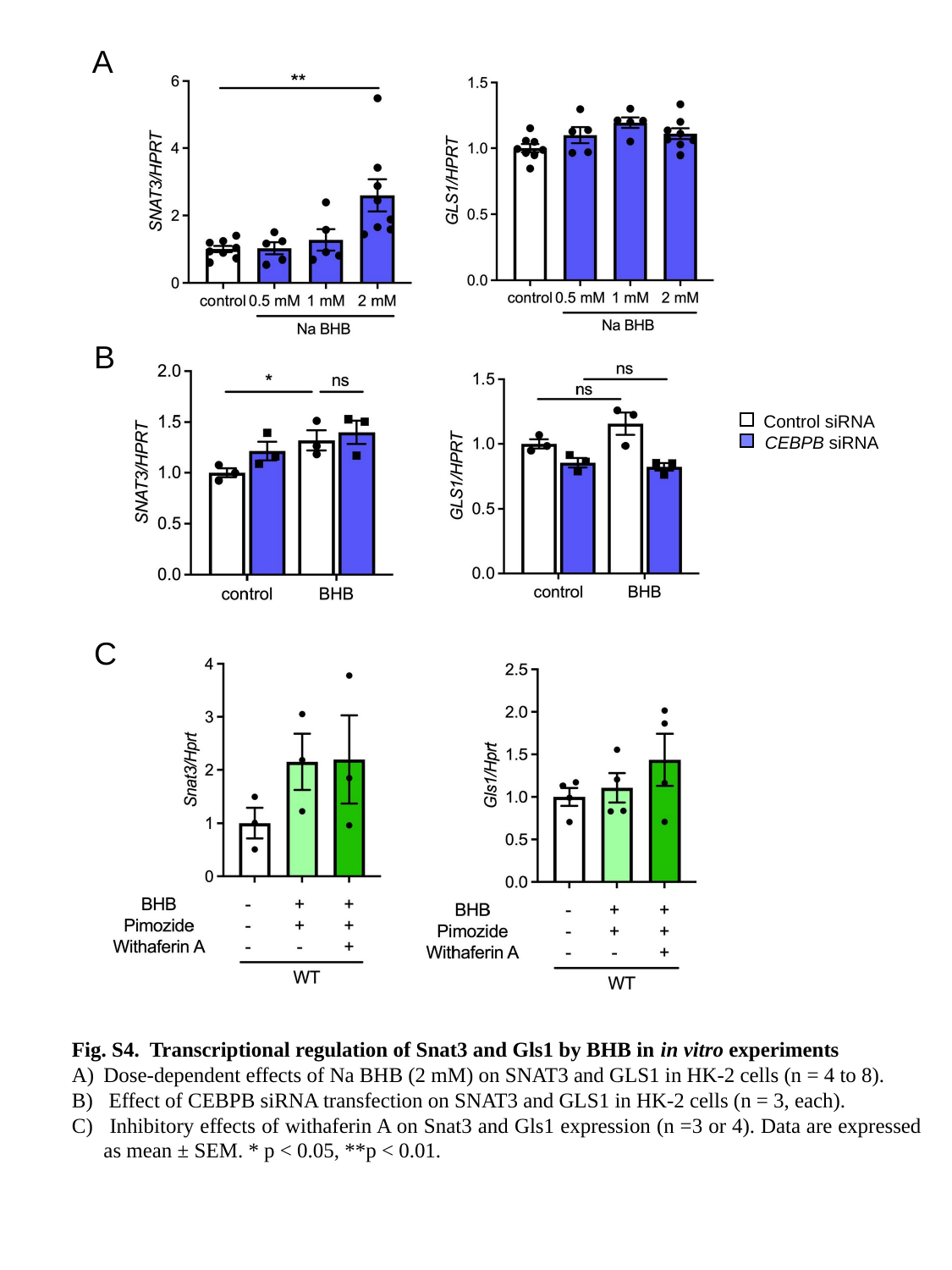

A
B
Control siRNA
CEBPB siRNA
C
Fig. S4. Transcriptional regulation of Snat3 and Gls1 by BHB in in vitro experiments
Dose-dependent effects of Na BHB (2 mM) on SNAT3 and GLS1 in HK-2 cells (n = 4 to 8).
 Effect of CEBPB siRNA transfection on SNAT3 and GLS1 in HK-2 cells (n = 3, each).
 Inhibitory effects of withaferin A on Snat3 and Gls1 expression (n =3 or 4). Data are expressed as mean ± SEM. * p < 0.05, **p < 0.01.
