# Supplementary material for: Hepatic ketone body regulation of renal gluconeogenesis
Source: Mol Metab. 2024 Apr 9;84:101934. doi: 10.1016/j.molmet.2024.101934 (PMC11039402; doi:10.1016/j.molmet.2024.101934)
Supplement: Multimedia component 2 [file mmc2.docx]

**Supplementary Materials**

**Methods**

***Immunofluorescent analysis***

Mouse kidney was fixed with 4% paraformaldehyde in 0.1 mol/L phosphate buffer (pH 7.4), embedded in paraffin and then cut into thin sections (4 μm). After deparaffinization and rehydration, the sections were immersed in a 0.01-mol/L citrate buffer and autoclaved for activation of immunogenicity. Slides were then washed in PBS and incubated with primary antibodies overnight at 4 °C. Next, slides were washed with PBS-T (0.1% Tween 20 in PBS) and then incubated with the secondary antibodies for 1 hr at room temperature. After a final PBS-T wash, the sections were mounted with fluorescent mounting medium (DAKO, Kyoto, Japan) and examined using a confocal laser scanning microscope (FV-1000D IX-81, Olympus, Tokyo, Japan).

***Western blot analysis***

Mice were sacrificed and the renal cortex was rapidly excised. Each tissue was homogenized in RIPA buffer followed by centrifugation at 3000 xg for 10min to collect total lysates. Five μg of protein was loaded into each lane for Laemmli’s sodium dodecyl sulfate–polyacrylamide gel electrophoresis (10–12.5%) and then transferred to a polyvinylidendifluoride membrane. The membrane was blocked for 1 hr by using 2.5% milk powder in TBST (10 mmol/l Tris-HCl, pH 8.5, 150 mmol/l NaCl, and 0.1% Tween 20) and was exposed to primary antibodies diluted with Solution 1 (Can Get Signal; TOYOBO Inc, Japan) overnight at 4 °C. After a TBST rinse, the secondary antibody diluted with Solution 2 (Can Get Signal; TOYOBO Inc, Japan) was applied to the membrane for 1 hr at room temperature. After washing, antigen–antibody complexes were visualized with a chemiluminescence system (Immobilon, Merck Millipore, MA, US).

***RNA-sequencing***

RNA integrity was checked by Agilent TapeStation 2200. RNA sequencing libraries were generated using the SMART-seq v4 Ultra Low RNA input kit (TaKaRa Bio Inc., Shiga, Japan) and purified using AMPure XP (Beckman Coulter Inc., CA). The quality of the cDNA libraries was evaluated by electrophoresis. The Illumina NovaSeq 6000 platform (Illumina, CA) was used to generate 150-bp paired-end sequencing reads. Base calling was performed using Real Time Analysis software (v3.4.4, Illumina, CA) and data were demultiplexed and converted to the FASTQ format using bcl2fastq2 software (v2.20, Illumina). Differentially expressed genes (DEGs) were determined by comparing the difference between the log2 transcripts per million (TPM) values of PTs from vehicle- or 1,3-BD-treaed mice (n = 4, each), which was statistically evaluated with a t-test. DAVID v6.8 (https://david.ncifcrf.gov) was used to investigate the enrichment of Gene Ontology (GO) and Kyoto Encyclopedia of Genes and Genomes (KEGG) pathways for the differentially expressed genes.

**Supplementary Tables**

**Table S1.**

**List of primers used in this study**

| **gene** | **Forward primer** | **Reverse primer** |
| --- | --- | --- |
| mHprt | 5′-GCGTCGTGATTAGCGATGA-3′ | 5′-ATGGCCTCCCATCTCCTT-3′ |
| mG6pc1 | 5′-GTGGCAGTGGTCGGAGACT -3′ | 5′-ACGGGCGTTGTCCAAAC-3′ |
| mPck1 | 5′-CACCATCACCTCCTGGAAGA-3′ | 5′-GGGTGCAGAATCTCGAGTTG -3′ |
| mSnat3 | 5′-GGCATATTTGGGATCATTGG-3′ | 5′-CCAGGATTTTAGGGGTGGAT-3′ |
| mGls1 | 5′-GGCAAAGGCATTCTATTGGA-3′ | 5′-TTGGCTCCTTCCCAACATAG-3′ |
| mHmgcs2 | 5′-AAACTTCGCTCACACCTGCT-3′ | 5′-AAGGATGCCCACATCTTTTG-3′ |
| mCebpb | 5′-GGGTTGTTGATGTTTTTGG-3′ | 5′-CGAAACGGAAAAGGTTCTCA-3′ |
| hHPRT | 5′-GACCAGTCAACAGGGGACAT-3′ | 5′-CCTGACCAAGGAAAGCAAAG-3′ |
| hG6PC1 | 5′-TGAGGATGGAGGAAGGAATG -3′ | 5′-GGGGAAGAGGACGTAGAAGG-3′ |
| hPCK1 | 5′-AGGCGGCTGAAGAAGTATGA-3′ | 5′-GGATGGGCACTGTGTCTCTT-3′ |
| hCEBPB | 5′-TTTCGAAGTTGATGCAATCG-3′ | 5′-CAACAAGCCCGTAGGAACAT-3′ |

m: mouse, h: human

**Table S2.**

**List of primary antibodies used in this study**

| **Antigen** | **Host** | **Clonality** | **Supplier** | **Product #** | **dilution** |
| --- | --- | --- | --- | --- | --- |
| PCK1 | rabbit | polyclonal | Abcam | ab70358 | 1:100(IF),1:1000(WB) |
| β-actin | rabbit | polyclonal | CST | #4967 | 1:1000(WB) |
| Villin (1D2C3) | mouse | monoclonal | Santa Cruz | sc-58897 | 1:200 (IF) |
| E-cadherin | mouse | monoclonal | BD | 610182 | 1:200 (IF) |
| THP (G-20) | goat | polyclonal | Santa Cruz | sc-19554 | 1:200 (IF) |
